# ISGylation of EMD promotes its interaction with PDHA to inhibit aerobic oxidation in lung adenocarcinoma

**DOI:** 10.1111/jcmm.17536

**Published:** 2022-09-07

**Authors:** Congcong Zhang, Jiangtao Cui, Leiqun Cao, Xiaoting Tian, Yayou Miao, Yikun Wang, Shiyu Qiu, Wanxin Guo, Lifang Ma, Jinjing Xia, Xiao Zhang

**Affiliations:** ^1^ Anhui University of Science and Technology School of Medicine Huainan Anhui China; ^2^ Department of Thoracic Surgery, Shanghai Chest Hospital Shanghai Jiao Tong University School of Medicine Shanghai China; ^3^ Shanghai Institute of Thoracic Oncology, Shanghai Chest Hospital, Shanghai Jiao Tong University School of Medicine Shanghai China; ^4^ Department of Clinical Laboratory Medicine Shanghai Chest Hospital, Shanghai Jiao Tong University School of Medicine Shanghai China; ^5^ Department of Pulmonary Medicine Shanghai Chest Hospital, Shanghai Jiao Tong University School of Medicine Shanghai China

**Keywords:** glucose metabolism, glycolysis, ISG15, proteasome, skeletal proteins, ubiquitination

## Abstract

Abnormal nuclear structure caused by dysregulation of skeletal proteins is a common phenomenon in tumour cells. However, how skeletal proteins promote tumorigenesis remains uncovered. Here, we revealed the mechanism by which skeletal protein Emerin (EMD) promoted glucose metabolism to induce lung adenocarcinoma (LUAD). Firstly, we identified that EMD was highly expressed and promoted the malignant phenotypes in LUAD. The high expression of EMD might be due to its low level of ubiquitination. Additionally, the ISGylation at lysine 37 of EMD inhibited lysine 36 ubiquitination and upregulated EMD stability. We further explored that EMD could inhibit aerobic oxidation and stimulate glycolysis. Mechanistically, via its β‐catenin interaction domain, EMD bound with PDHA, stimulated serine 293 and 300 phosphorylation and inhibited PDHA expression, facilitated glycolysis of glucose that should enter the aerobic oxidation pathway, and EMD ISGylation was essential for EMD‐PDHA interaction. In clinical LUAD specimens, EMD was negatively associated with PDHA, while positively associated with EMD ISGylation, tumour stage and diameter. In LUAD with higher glucose level, EMD expression and ISGylation were higher. Collectively, EMD was a stimulator for LUAD by inhibiting aerobic oxidation via interacting with PDHA. Restricting cancer‐promoting role of EMD might be helpful for LUAD treatment.

## 1 INTRODUCTION

Skeletal proteins are the main support structures of cells.^1^ In addition to its important role in the stability of cell morphology, and the ability to withstand external forces and the orderliness of internal structures, it also participates in tumorigenesis.^2^, ^3^ The cytoskeleton may induce cell proliferation through the activity of specific proteins that control cell stiffness, and oncogenes become active throughout this process.^4^ Alterations in cytoskeletal proteins also lead to nuclear abnormalities common in a wide range of cancer types.^5^ It has long been recognized that tumour cells frequently exhibit abnormal nuclear architecture, including nuclear size and structure, nucleolar size and number and chromatin texture, and that these alterations are critical risk factors for tumour formation and developments.^6^, ^7^

Emerin (EMD) is 254 amino acids in length and with an N‐terminal globular LAP2‐EMD‐MAN1 (LEM, aa: 1–45) domain, followed by an β‐catenin interaction (IT, aa: 168–186) domain,^8^ and anchored to the nuclear envelope.^9^ EMD is an integral nuclear membrane protein that is expressed in most human tissues and is involved in the maintenance of nuclear structure.^9^, ^10^ EMD also has important effects on nuclear assembly cell cycle progression.^11^ Furthermore, EMD has been implicated in the regulation of gene expression, cellular signalling, and nuclear and genomic structure.^11^ Although EMD was highly expressed in various types of cancers including ovarian epithelial cancer^12^ and breast cancer,^13^ how EMD exerts its ontogenetic role in cancer progression remain largely uncovered.

Interferon‐stimulated gene 15 (ISG15) is a highly inducible interferon‐stimulated gene first discovered by Haas et al.^14^ ISG15 covalently binds to the target protein through the glycine–glycine motif at the C‐terminus and modifies the target protein. This binding is called ISGylation. ISGylation is similar to ubiquitination, but it does not function to degrade proteins. It is closely related to stress response, transcriptional regulation and protein translation.^15^ On the contrary, ISGylation could be an antagonistic process for ubiquitination and the following degradation via proteasomes. For example, through in vitro studies, it is known that ISG15 and ubiquitin (Ub) can form mixed chains. Lysine (K) residues 29 and 48 on Ub are the substrates for ISG15, and ISG15 may alter the binding of Ub to its target proteins and inhibit their subsequent degradation.^16^ Moreover, an increase in the ubiquitination levels in breast cancer cells is promoted by the reduction of ISG15 or UBCH8 (ubiquitin‐conjugating enzyme H8, an ISG15 conjugating E2 enzyme), suggesting that ISGylation avoids protein ubiquitination and confers stability.^17^ In other studies, it has been demonstrated that the ISGylation of the interferon regulatory factor 3 (IRF3) transcription factor decreases its interaction with peptidylprolyl cis/trans isomerase, NIMA‐interacting 1 (PIN1) to avoid its ubiquitination.^18^ However, whether the protein ISGylation can inhibit ubiquitination in other ways needs further elucidation.

Glucose metabolism is usually abnormal in tumour. Aerobic oxidation is inhibited, while glycolysis, pentose phosphate pathway and hexosamine biosynthesis pathway (HBP) are activated.^19^, ^20^ Glycolysis, pentose phosphate and HBP pathways produce less adenosine triphosphate (ATP), but can provide more internal products for tumour cell proliferation compared with aerobic oxidation.^21^ Catalytic alpha subunit of PDHc (PDHA) is an enzyme that acts as a key link between glycolysis and the tricarboxylic acid cycle, converting acetyl coenzyme A (acetyl‐CoA) to pyruvate. PDHA is phosphorylated and inhibited by pyruvate dehydrogenase kinase (PDK).^22^ Studies have pointed out that abnormal PDHA expression is closely related to the occurrence and development of various tumours, including glioma,^23^ cholangiocarcinoma,^24^ breast cancer^25^ and so on. However, whether PDHA links with lung adenocarcinoma (LUAD) and its relationship with skeletal proteins in tumour formation and development remain elusive.

Here, we identified that EMD was highly expressed in LUAD and stimulated LUAD malignant phenotypes. In addition, the ubiquitination level of EMD was lower, but its stability was higher than that of other skeleton proteins. EMD ISGylation inhibited its ubiquitination and the following proteasome degradation. Moreover, we noticed that EMD promoted glucose uptake in LUAD cells and inhibited aerobic oxidation, allowing glucose to enter the glycolysis pathway. Mechanistically, EMD IT domain was essential for interaction and inhibition to PDHA expression and phosphorylation. In clinical LUAD specimens, EMD was negatively associated with PDHA, while positively associated with EMD ISGylation, tumour stage and diameter. LUAD with higher glucose level was associated with higher EMD expression and ISGylation level compared with that with lower glucose level. Therefore, EMD was a stimulator for glucose metabolism in LUAD, new treatment strategies for LUAD could be proposed considering the impact of EMD and its ISGylation.

## 2 MATERIAL AND METHODS

### 2.1 Cell culture

A549, H1299, PC9, Calu‐1, H1975 and H2030 cell lines were purchased from Fuheng Biotechnology (Shanghai, China). All cells were maintained in Dulbecco's modified eagle's medium (DMEM, HyClone, Logan, UT, USA) supplemented with 10% foetal bovine serum (FBS), 100 U/ml penicillin and 100 mg/ml streptomycin.

### 2.2 Mouse experiments and tissue samples

For xenograft experiments, H2030 cells (initially 5 × 10^6^) with EMD^WT‐FLAG^ or EMD^Del‐IT‐FLAG^ overexpression with or without ISG15 knockout were subcutaneously injected into 8‐week‐old athymic nude mice. For patient‐derived xenograft (PDX) generation, fresh LUAD tissues in the size of 2–3 mm^3^ were subcutaneously implanted into 8‐week‐old athymic nude mice. The PDX‐planted mice could be used for further study after successful passage. The tumour volume was calculated as 0.5 × L × W^2^, with L indicating length and W indicating width. All the tissue samples (mean age ± SD, 61.16 ± 10.35 years; male: female ratio, 1.14:1) were recruited in Shanghai Chest Hospital (Shanghai, China) from May 2013 to January 2022. Cohort #1 were collected from March 2015 to May 2017. Cohort #2 were collected from January 2021 to May 2021. Cohort #3 were collected from May 2021 to July 2021. Cohort #4 were collected from May 2021 to September 2021. Cohort #5 were collected from May 2013 to March 2019. Cohort #6 were collected from September 2021 to January 2022. Cohort #1 was stored in the form of tissues slices in 4°C. Cohort #5 was stored in the form of tissue microarrays in 4°C. Cohort #2, #3, #4 and #6 samples were stored in the form of tissue blocks in −80°C. Other detailed information was summarized in Table S1‐6.

### 2.3 Regents and plasmids

For regents, cycloheximide (CHX, Sigma, St Louis, MO) and MG132 (MedChemExpress, Monmouth Junction, NJ) were used to treat LUAD cells.

For plasmids, ISG15 and ISG15‐KO plasmids were acquired from previous studies.^26^ EMD^WT‐FLAG^ and PDHA^WT‐HA^ were purchased from Zorinbio (Shanghai, China). lentiCRISPR v2‐based constructs were used for knocking out EMD. All the mutation plasmids were constructed using overlapping PCR and cloned into pcDNA3.1(+) plasmids (Biolink, Shanghai, China). The sequences for small guide RNA (sgRNA) were summarized in Table S7.

### 2.4 Immunofluorescence, immunochemistry, immunoblotting and enzyme linked immunosorbent assay

IF, immunochemistry and IB were performed according to the previous protocols.^27^, ^28^ For IF, anti‐EMD (Abcam, #ab156871), anti‐proteasome 20S subunit beta 5 (PSMB5, Abcam, #ab167341) and anti‐PDHA (Abcam, #ab110330) antibodies were used. For immunochemistry, anti‐EMD (Abcam, #ab156871) antibodies were used. IHC scores were computed by multiplying the staining intensity grade (0, 1, 2 and 3 represented negative, weak‐positive, moderate‐positive and strong‐positive, respectively) by the positive rate score (0, 1, 2, 3 and 4 represented positive areas of ≤5%, 6%–25%, 26%–50%, 51%–75%, and ≥ 76%, respectively) as we previously described.^29^ For IB, anti‐EMD (Abcam, #ab40688 and #ab204987), anti‐Actin (Abcam, #ab6276), anti‐Lamin A (Abcam, #ab8980), anti‐Nesprin1 (Abcam, #ab192234), anti‐Nesprin3 (Abcam, #ab186746), anti‐SUN domain‐containing protein 1 (Sun1, Abcam, #ab124770), anti‐Sun2 (Abcam, #ab124916), anti‐glyceraldehyde‐3‐phosphate dehydrogenase (GAPDH, Abcam, #ab9485 and #ab8245), anti‐Ub (Abcam, #ab134953 and #ab7254), anti‐FLAG (CST, Boston, MA, USA #14793 and #8146), anti‐PSMB5 (Abcam, #ab3330), anti‐aldolase (ALDO, Abcam, #ab150396), anti‐enolase 1 (ENO1, Abcam, #ab227978), anti‐glucose‐6‐phosphate isomerase (GPI, Abcam, #ab167394), anti‐hexokinase (HK, Abcam, #ab150423), anti‐lactate dehydrogenase (LDH, Abcam, #ab52488), anti‐PDHA (Abcam, #ab168379 and #ab110330), anti‐PDHB (Abcam, #ab155996), anti‐pan‐phosphoinositide‐dependent protein kinase (PDK, Abcam, #ab115321), anti‐phosphofructokinase (PFK, Abcam, #ab240237), anti‐phosphoglycerate mutase 1 (PGAM1, Abcam, #ab129191), anti‐phosphoglycerate kinase (PGK, Abcam, #ab186742), anti‐pyruvate kinase M1/2 (PKM, Abcam, #ab150377), anti‐HA (Abcam, #ab9110 and #ab1424), anti‐3‐phosphoinositide‐dependent protein kinase 1 (PDK1, Abcam, #ab202468) and anti‐ISG15 (Abcam, #ab285367) antibodies were used. The relative protein levels were normalized to those of internal reference protein as calculated by ImageJ software and indicated just below the blots. The original blots were summarized in Supplementary Materials. For ELISA, EMD and PDHA levels were measured using kits from Yingxin Biotech Ltd. (Shanghai, China) as per manufacturer's instructions.

### 2.5 Co‐immunoprecipitation

Co‐immunoprecipitation was performed as described previously.^27^, ^28^, ^30^, ^31^ Cell lysates were mixed with protein A/G‐magnetic beads (Novex, Oslo, Norway) and incubated at 4°C overnight with the selected antibodies. The beads were washed using Western/IP lysis buffer (Beyotime, Haimen, China, composition: 20 mM Tris [pH 7.5], 150 mM NaCl, 1% Triton X‐100, and inhibitors containing sodium pyrophosphate, β‐glycerophosphate, EDTA, Na_3_VO_4_ and leupeptin), and eluted using the Acid Elution buffer (Beyotime). Next, total protein amount of each group was measured by BCA protein quantification. After the first quantification, the samples were diluted according to the difference in protein amount, and the second quantification was carried out for confirmation. Finally, the samples were suspended in SDS‐PAGE loading buffer and then measured by IB. The antibodies used for co‐IP were as follows: anti‐FLAG (CST, #14793 and #8146), anti‐PDHA (Abcam, #ab168379 and #ab110330), anti‐HA (Abcam, #ab9110 and #ab1424), anti‐EMD (Abcam, #ab40688 and #ab204987), anti‐Lamin A (Abcam, #ab226198), anti‐Nesprin1 (Abcam, #ab192234), anti‐Nesprin3 (Abcam, #ab186746), anti‐Sun1 (Abcam, #ab124770) and anti‐Sun2 (Abcam, #ab124916).

### 2.6 Measurements of cell viability and anchorage‐independent colony formation

Cell viability was measured using a 3‐(4,5‐dimethylthiazol‐2‐yl)‐2,5‐diphenyltetrazolium bromide (MTT)‐dependent method. As for colony formation assay, LUAD cells were seeded in a 6‐well plate containing 0.3% agarose in DMEM at a density of 6 × 10^3^ cells per well. After 2 weeks, the numbers of colonies were counted under microscope.

### 2.7 Quantitative RT‐PCR

Total RNA was extracted using Trizol‐ (Ambion, Carlsbad, CA, USA) dependent method and reverse‐transcribed into complementary DNA using the PrimeScript™ RT reagent kit (Takara, Dalian, China). Next, the SYBR premix Ex Taq (Takara) kit was used for real‐time qPCR. The primers are listed in Table S7.

### 2.8 Proteasome isolation

Proteasomes were isolated by proteasome isolation kit (Sigma, #539176). Isolation was performed in accordance with manufacturer guidelines. Affinity and control beads were used to isolate the proteasome and serve as a negative control, respectively.

### 2.9 Extracellular acidification rate and oxygen consumption rate assays

ECAR and OCR were analysed using the extracellular flux analyser XF96 (Seahorse Bioscience, Billerica, MA, USA) with the glycolysis stress test kit (Agilent, Wilmington, DE, USA, # 103020–100) and mitochondrial stress test kit (Agilent, # 103015–100), respectively.

### 2.10 Measurements of metabolites and O‐GlcNAcylated proteins

Glucose, acetyl‐CoA, lactic acid, citrate, isocitrate, α‐ketoglutarate, succinate, fumarate, malate, oxaloacetate, pyruvate and phosphoenolpyruvate (PEP, upstream of pyruvate) concentrations were measured by kits purchased from Sigma. 6‐phosphogluconate (6‐PG) concentration was measured by kit purchased from Abcam. ribose‐5‐phosphate (Rib‐5‐P) and uridine diphospho‐N‐acetylglucosamine (UDP‐GlcNAc) concentrations were measured by kits purchased from Yingxin. O‐GlcNAcylated proteins were measured using the kits from Abcam. All the experiments were performed strictly according to the instruction provided by the manufacturer.

### 2.11 Protein ligation assay experiments

PLA was performed to identify the direct interactions between two proteins using the Duolink in situ Red Starter Kit (mouse/rabbit) (Sigma). The detailed procedure was described in a previous study.^28^ The antibodies used were anti‐EMD (Abcam, #ab156871) and anti‐PDHA (Abcam, #ab110330).

### 2.12 Statistical analysis

Tests for this study included Student's *t*‐test, one‐way, two‐way anova, χ^2^ test and the Spearman rank‐correlation analysis. A *P* < 0.05 was considered statistically significant.

### 2.13 Ethics statement

This study was approved by the Ethics Review Committee of Shanghai Chest Hospital, Shanghai Jiao Tong University School of Medicine (permit number: KS[Y]1922). All patients completed informed consent forms.

## 3 RESULTS

### 3.1 EMD protein was highly expressed in LUAD

**FIGURE 1:**
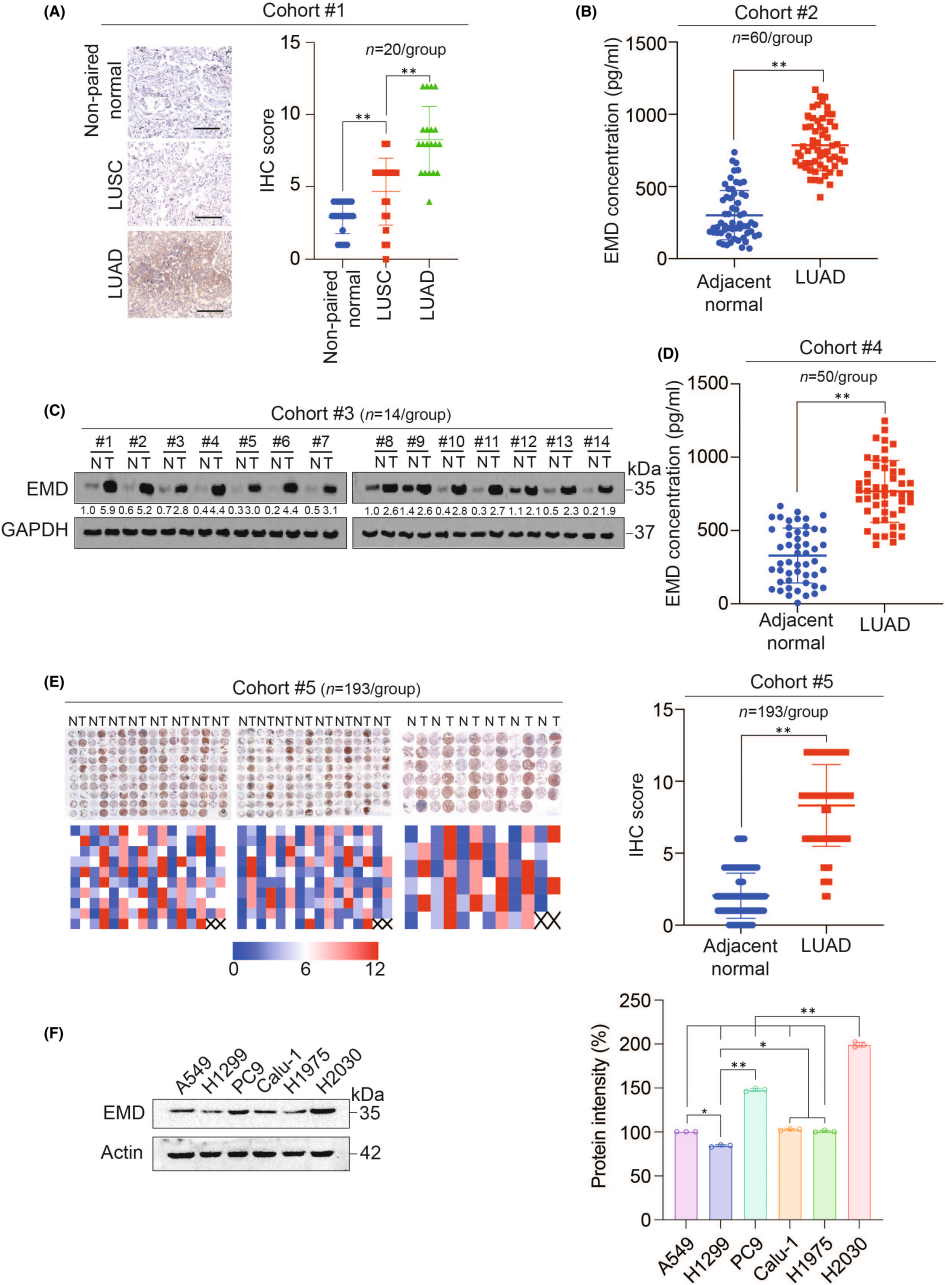
EMD protein was highly expressed in LUAD. (A) EMD protein expression was measured by IHC in cohort #1 (including 20 LUSC tissues, 20 LUAD tissues and 20 randomly selected non‐paired tumour‐adjacent normal tissues). IHC score was calculated. Scale bar, 500 μm. (B) EMD protein concentration was measured by ELISA in cohort #2 (including 60 LUAD tissues and their adjacent normal tissues). (C) EMD protein expression was measured by IB in cohort #3 (including 14 LUAD tissues and their adjacent normal tissues). (D) EMD protein level was measured by ELISA in cohort #4 (including 50 LUAD tissues and their adjacent normal tissues). (E) EMD protein expression was measured by IHC in cohort #5 (TMA including 193 LUAD tissues and their adjacent normal tissues). IHC score was calculated. (F) EMD protein expression was measured by IB in LUAD cell lines including A549, H1299, PC9, Calu‐1, H1975 and H2030. Protein intensity was calculated. IB images were selected from three biological replicates. Data in A and F were analysed using a one‐way anova test. Data in B, D and E were analysed using a Students' *t*‐test. *, *P* < 0.05, **, *P* < 0.01

Firstly, we explored EMD expression in LUAD by analysing five cohorts. In cohort #1 (including 20 lung squamous cell carcinoma [LUSC] tissues, 20 LUAD tissues and 20 normal tissues adjacent to tumour), we found that the expression intensity of EMD in normal tissues was lower than that in LUSC tissues, which in turn was lower than that in LUAD tissues (Figure 1A). After analysing tissues from cohort #2 (including 60 paired of LUAD and adjacent normal tissues), cohort #3 (including 14 paired of LUAD and adjacent normal tissues) and cohort #4 (including 50 paired of LUAD and adjacent normal tissues), we found that EMD protein was highly expressed in LUAD tissues compared with adjacent normal tissues, whereas EMD mRNA level was not changed between LUAD and adjacent normal tissues (Figure 1B‐D and Figure S1A‐C). Additionally, in cohort #5 (including 193 paired of LUAD and adjacent normal tissues), we also observed that EMD expression was higher in LUAD tissues compared with adjacent normal tissues (Figure 1E). These data suggested that EMD protein expression was elevated in LUAD. We further screened six LUAD cell lines and selected the cell line with highest EMD expression (H2030) and lowest EMD expression (H1299) (Figure 1F and Figure S1D‐E).

### 3.2 EMD was hypoubiquitinated in LUAD cells

**FIGURE 2:**
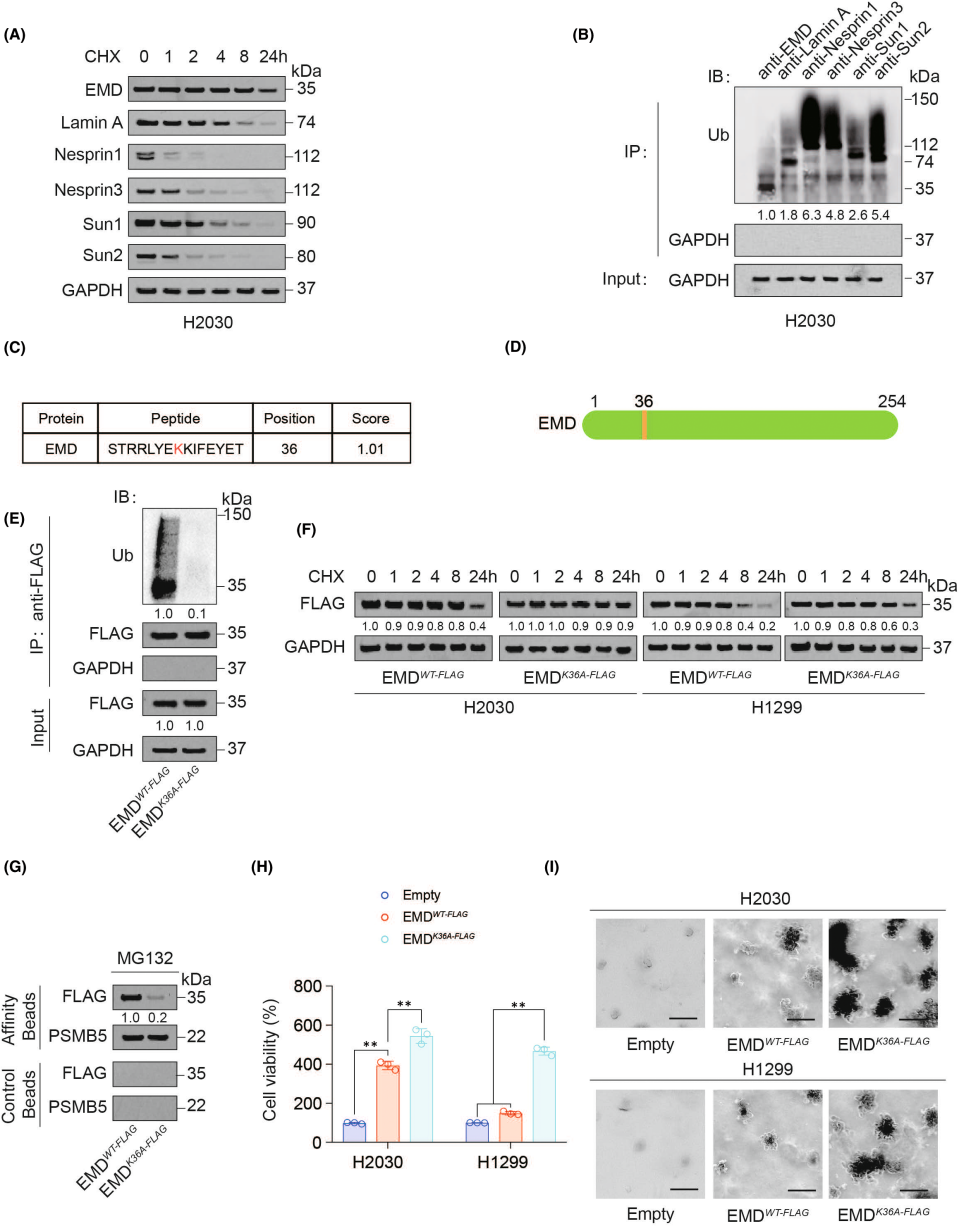
EMD was hypoubiquitinated in LUAD. (A) Skeletal protein levels were measured by IB in H2030 cells treated with CHX (10 μg/ml) for indicated hours. (B) Ubiquitination of indicated proteins were measured by co‐IP using their antibodies followed by IB in H2030 cells. The total protein level in each co‐IP sample was adjusted to the same protein content. (C) EMD ubiquitination site predicted by BDM‐PUB database. (D) The location of the predicted EMD ubiquitination site on total protein. (E) The co‐IP experiment was performed using anti‐FLAG antibodies in H2030 cells with EMD^WT‐FLAG^ or EMD^K36A‐FLAG^ overexpression. EMD‐FLAG ubiquitination was measured by IB. (F) EMD^WT‐FLAG^ or EMD^K36A‐FLAG^ expression was measured by IB in H2030 or H1299 cells with CHX (10 μg/ml) treatment for indicated hours. (G) Association of EMD and PSMB5 analysed by IB in proteasomes isolated from EMD^WT‐FLAG^ or EMD^K36A‐FLAG^ overexpressed H2030 cells in the presence of MG132 (8 μM, 24 h). Samples from affinity or control beads were analysed in parallel. (H‐I) Cell viability (H) and colony formation (I) were measured in H2030 and H1299 cells with or without EMD^WT‐FLAG^ or EMD^K36A‐FLAG^ overexpression. Scale bar, 100 μm. The data are shown as the mean ± SD from three biological replicates (including IB). Data in H were analysed using a one‐way anova test. **, *P* < 0.01

Since EMD protein level was elevated in LUAD while mRNA level was unchanged, we ruled out the possibility that RNA modification and transcription contributed to the EMD elevation. Ubiquitination is a mechanism that inhibits protein expression by reducing protein stability.^32^, ^33^ We hypothesized that the increased expression of EMD in LUAD might be related to its ubiquitination status. We found that the stability of EMD was higher than that of several other common cytoskeleton proteins, including Lamin A, Nesprin 1, Nesprin 3, Sun1, Sun2, in either the H2030 cell line with highest EMD expression or the H1299 cell line with lowest EMD expression (Figure 2A and Figure S2A‐C). In H2030 cells, compared with other cytoskeleton proteins, EMD had the lowest ubiquitination level (Figure 2B and Figure S2D‐E). These data suggested that high expression of EMD might be because of its hypoubiquitinated station.

### 3.3 EMD was ubiquitinated in K36

We used Prediction of Ubiquitination sites with Bayesian Discriminant Method (BDM‐PUB) database (http://bdmpub.biocuckoo.org/prediction.php) to predict potential EMD ubiquitinated sites, and found its K36 position has the highest possibility of being modified (Figure 2C‐D). After K36 was mutated to alanine (A), the ubiquitination of EMD was significantly decreased (Figure 2E), resulting in an increase in the stability of EMD (Figure 2F) and a significant decrease in the ability of EMD to enter the proteasome (Figure 2G). In addition, K36A mutation of EMD enhanced cell viability and colony formation ability of H2030 and H1299 cells (Figure 2H‐I and Figure S2F). These data suggested that K36 was an important ubiquitination site for EMD.

### 3.4 EMD ISGylation inhibited its ubiquitination to increase protein stability

**FIGURE 3:**
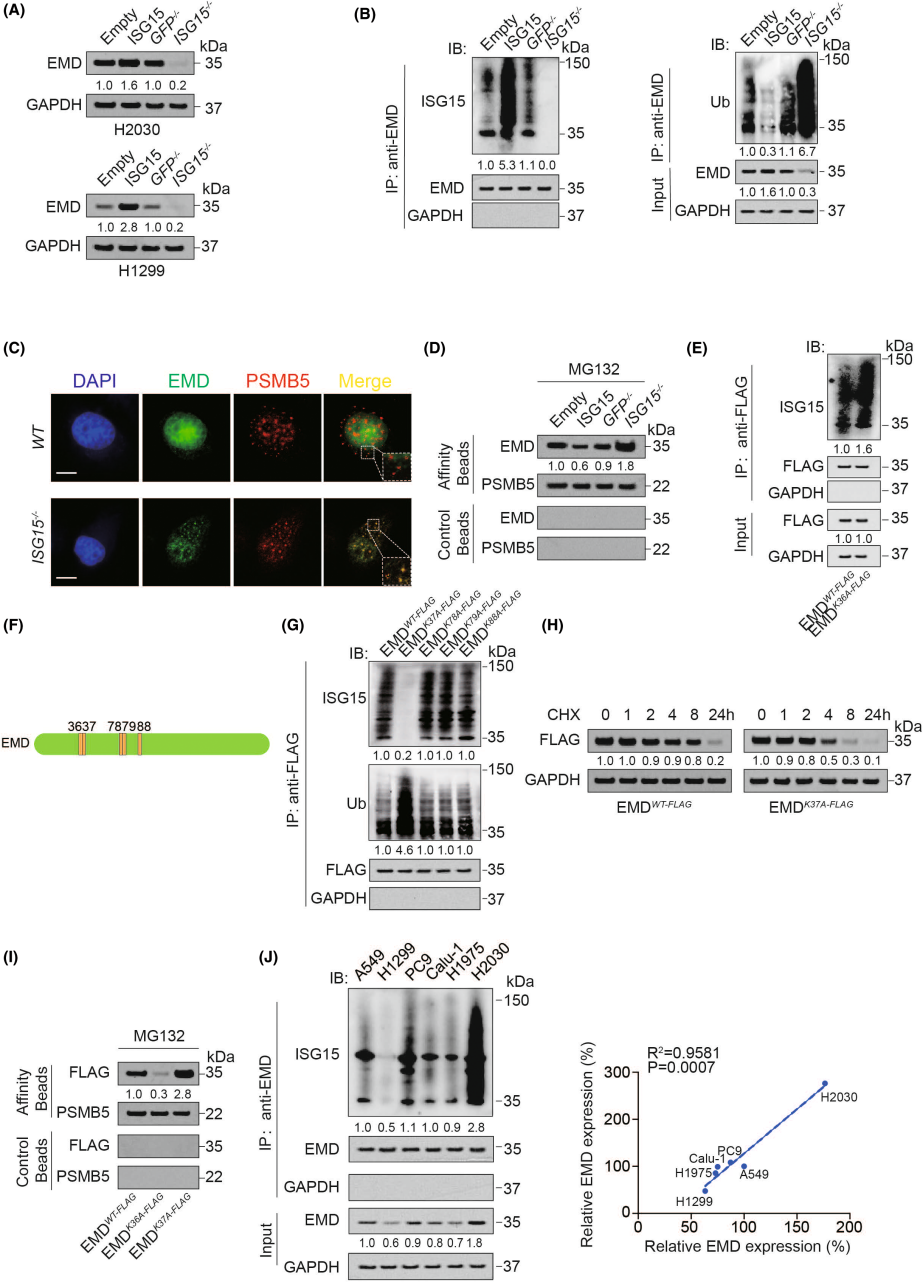
EMD ISGylation inhibited ubiquitination. (A) EMD expression in ISG15 overexpression or knockout H2030 and H1299 cells as measured by IB. (B) EMD ISGylation and ubiquitination were measured in H2030 cells with or without EMD overexpression or knockdown. The EMD level in each co‐IP sample was adjusted to the same protein content. (C) EMD and PSMB5 colocalization in *GFP* ^*−/−*^ or *ISG15* ^*−/−*^ H2030 cells was measured by IF. Scale bar, 20 μm. (D) Association of EMD and PSMB5 analysed by IB in proteasomes isolated from ISG15 overexpression or knockout H2030 cells in the presence of MG132 (8 μM, 24 h). Samples from affinity or control beads were analysed in parallel. (E) ISGylation of EMD^WT‐FLAG^ and EMD^K36A‐FLAG^ were measured in H2030 cells. (F) The location of the potential EMD ISGylation site on total protein. (G) ISGylation and ubiquitination of EMD^WT‐FLAG^, EMD^K37A‐FLAG^, EMD^K78A‐FLAG^, EMD^K79A‐FLAG^ and EMD^K88A‐FLAG^ were measured in H2030 cells. (H) EMD^WT‐FLAG^ or EMD^K37A‐FLAG^ was measured by IB in H2030 cells with CHX (10 μg/ml) treatment for indicated hours. (I) Association of EMD and PSMB5 analysed by IB in proteasomes isolated from EMD^WT‐FLAG^, EMD^K36A‐FLAG^ or EMD^K37A‐FLAG^ overexpressed H2030 cells in the presence of MG132 (8 μM, 24 h). Samples from affinity or control beads were analysed in parallel. (J) Relationship between EMD ISGylation and expression were measured in indicated LUAD cell lines. The EMD level in each co‐IP sample was adjusted to the same protein content. IB images were selected from three biological replicates. Data in J were analysed by a Spearman rank‐correlation analysis

Since ISGylation and ubiquitination both occurs at K residue,^34^ we subsequently investigated whether ISGylation influenced EMD stability and expression via its ubiquitination. We found that in both H2030 and H1299 cells, overexpression of ISG15 increased, whereas knockout of ISG15 decreased EMD expression (Figure 3A). In addition, ISG15 elevated the ISGylation of EMD while inhibited its ubiquitination (Figure 3B and S3A). Knockout of ISG15 significantly promoted colocalization of EMD and one active site of the proteasome, PSMB5 (Figure 3C and S3B), and ISG15 also inhibited the ability of EMD to enter the proteasome (Figure 3D). Unfortunately, the mutation of ubiquitination site K36 into A did not inhibit the ISGylation of EMD, indicating that K36 is not the ISGylation site of EMD (Figure 3E and S3C). We mutated the Ks near K36 (including K37, K78, K79, K88) into A, and found that only K37A enhanced ubiquitination of EMD and inhibited its ISGylation (Figure 3F‐G and S3D). In addition, K37A significantly decreased the stability of EMD (Figure 3H). We also found the ability of EMD to enter the proteasome was weakened by K36A and strengthened by K37A (Figure 3I). Moreover, the ISGylation of EMD was positively correlated with its expression in LUAD cell lines (Figure 3J and S3E). Collectively, these data suggested that ISGylation at the K37 site of EMD inhibited its ubiquitination at the K36 site and upregulated EMD protein stability.

### 3.5 EMD inhibited aerobic oxidation and stimulates glycolysis

**FIGURE 4:**
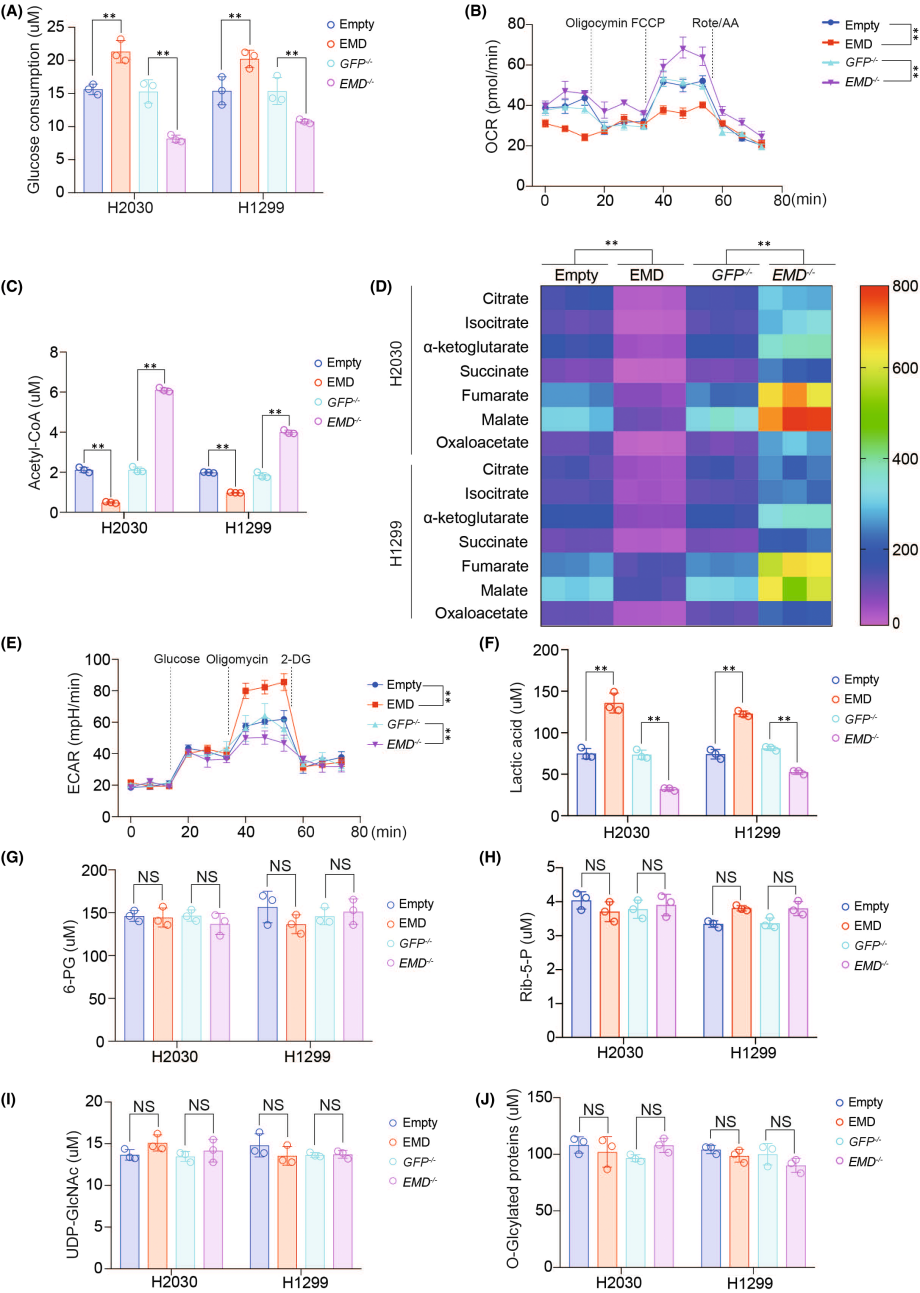
EMD inhibited aerobic oxidation and stimulated glycolysis. (A) Glucose consumption was measured in H2030 and H1299 cells with or without EMD overexpression or knockout. (B‐C) OCR (B) and acetyl‐CoA level (C) were measured in H2030 and H1299 cells with or without EMD overexpression or knockout. (D) Citrate, isocitrate, α‐ketoglutarate, succinate, fumarate, malate and oxaloacetate level were measured in H2030 and H1299 cells with or without EMD overexpression or knockout. (E‐J) ECAR (E), lactic acid (F), 6‐PG (G), Rib‐5‐p (H), UDP‐GlcNAc level and O‐GlcNAcylated proteins were measured in H2030 and H1299 cells with or without EMD overexpression or knockout. The data are shown as the mean ± SD from three biological replicates. Data in A, C, F, G, H, I and J were analysed using a Student's *t*‐test. Data in B, D and E were analysed using a two‐way anova test. **, *P* < 0.01, NS, non‐significant

The above data have confirmed that EMD could promote the malignant phenotype of LUAD cells (Figure 2H‐I), indicating that EMD could promote LUAD^35^. Abnormal glucose metabolism is an important factor in tumour development.^19^ We observed that EMD overexpression induced, while EMD knockout suppressed glucose consumption and intracellular glucose level (Figure 4A and S4A‐B), and EMD inhibited the biomarkers of aerobic oxidation including OCR (Figure 4B), acetyl‐CoA, and products of tricarboxylic acid cycle (citrate, isocitrate, α‐ketoglutarate, succinate, fumarate, malate and oxaloacetate) (Figure 4C‐D). In addition, we found that EMD stimulated ECAR, the biomarker of glycolysis (Figure 4E) and increased lactic acid level (Figure 4F). However, EMD did not affect pentose phosphate pathway products 6‐PG and Rib‐5‐P (Figure 4G‐H), as well as HBP products UDP‐GlcNAc and O‐GlcNAcylated proteins (Figure 4I‐J). These data suggested EMD inhibited aerobic oxidation and stimulates glycolysis.

### 3.6 EMD interacted and degraded PDHA

**FIGURE 5:**
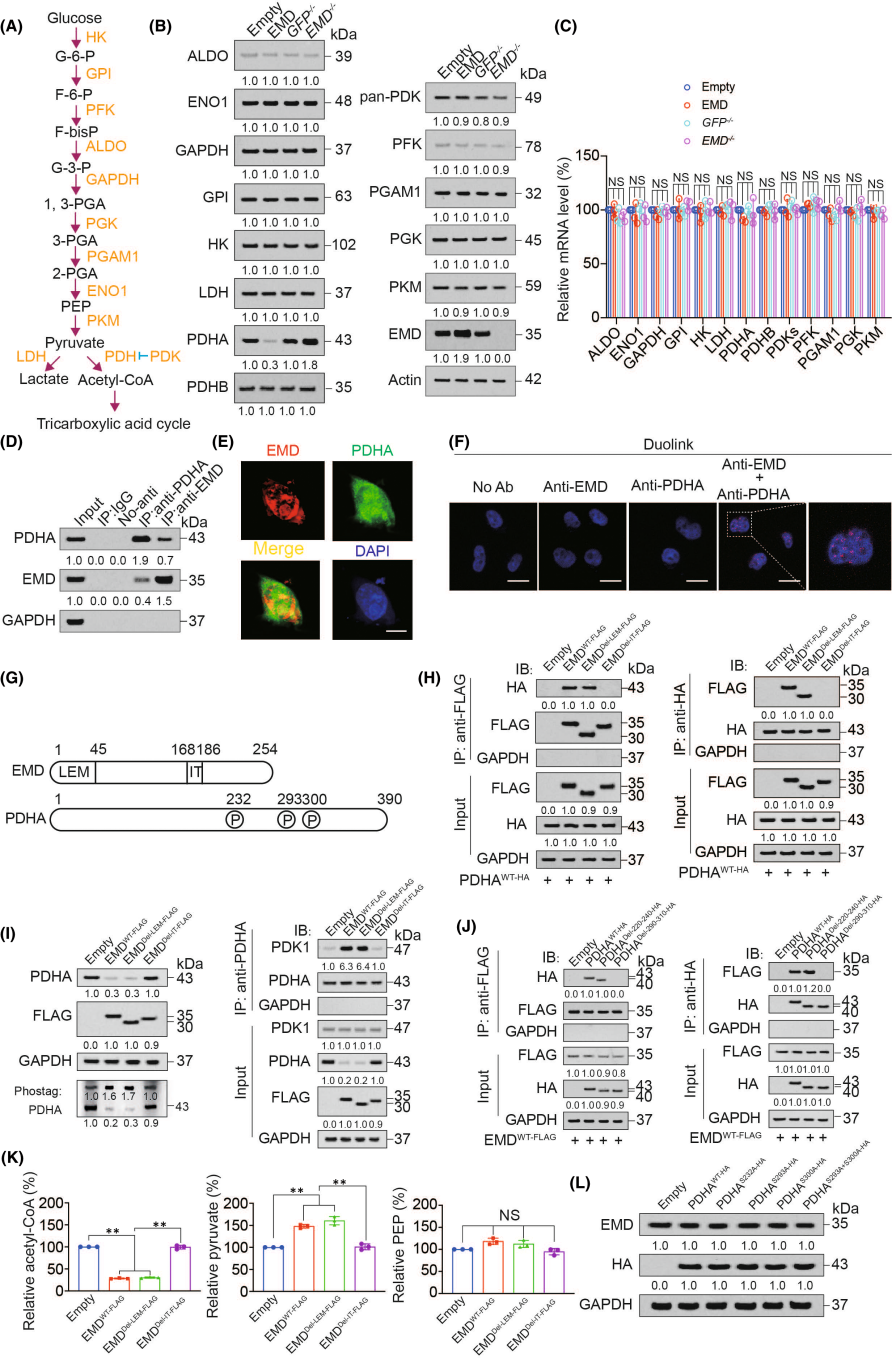
EMD interacted and inhibited PDHA. (A) Related metabolites and kinases in aerobic oxidation and glycolysis. (B‐C) Protein and mRNA level of aerobic oxidation and glycolysis related kinases were measured by IB (B) and qPCR (C) in EMD overexpressed or knockout H2030 cells. (D) Co‐IP experiments were performed using anti‐EMD or anti‐PDHA antibodies in H2030 cells. IgG‐immunoprecipitated or no‐antibody used samples were analysed in parallel. Co‐immunoprecipitated PDHA and EMD expression was measured. (E) Colocalization of EMD and PDHA was measured by IF in H2030 cells. Scale bar, 20 μm. (F) Proximal protein ligation between EMD and PDHA was measured by PLA in H2030 cells. Scale bar, 50 μm. (G) The location of the EMD IT and LEM domain and PDHA phosphorylation sites on their total proteins. (H) Reciprocal co‐IP experiments were performed using anti‐FLAG or anti‐HA antibodies in PDHA^WT‐HA^ overexpressed H2030 cells with or without EMD^WT‐FLAG^, EMD^Del‐LEM‐FLAG^ or EMD^Del‐IT‐FLAG^ overexpression. (I) PDHA expression and phosphorylation were measured by normal and Phostag‐IB, respectively, in H2030 cells with or without EMD^WT‐FLAG^, EMD^Del‐LEM‐FLAG^ or EMD^Del‐IT‐FLAG^ overexpression. PDHA and PDK1 interaction was measured by co‐IP using anti‐PDHA followed by IB experiments. The PDHA level in each co‐IP sample was adjusted to the same protein content. (J) Reciprocal co‐IP experiments were performed using anti‐FLAG or anti‐HA antibodies in EMD^WT‐FLAG^ overexpressed H2030 cells with or without PDHA^WT‐HA^, PDHA^Del‐220‐240‐HA^ or PDHA^Del‐290‐310‐HA^ overexpression. (K) Relative acetyl‐CoA, pyruvate and PEP level were measured in H2030 cells with or without EMD^WT‐FLAG^, EMD^Del‐LEM‐FLAG^ or EMD^Del‐IT‐FLAG^ overexpression. (L) EMD and HA expression were measured in H2030 cells with or without indicated WT or mutant PDHA overexpressed. The data are shown as the mean ± SD from three biological replicates (including IB). Data in C and K were analysed using a one‐way anova test. **, *P* < 0.01, NS, non‐significant

To further investigate how EMD regulated aerobic oxidation and glycolysis, we overexpressed or knocked out EMD to evaluate the effects of EMD on the expression of aerobic oxidation and glycolysis‐related metabolic enzymes including HK, GPI, PFK, ALDO, GAPDH, PGK1/2, PGAM1, ENO1, PKM, LDH, PDH and PDKs. We observed that EMD only affected the protein expression of PDHA but did not affect the protein expression of other enzymes (Figure 5B and S5A). In addition, EMD had no effect on mRNA expression of all enzymes (Figure 5C and S5B). EMD and PDHA protein colocalization and interaction were also validated (Figure 5D‐F and S5C). These data suggested that EMD bound to PDHA and inhibited PDHA expression.

### 3.7 Molecular mechanism for EMD‐PDHA interaction

EMD protein has two key protein interaction domains LEM and IT, and PDHA protein has three important phosphorylation sites serine (S) 232, S293 and S300 which are phosphorylated by PDK1 (Figure 5G).^8^, ^22^ Deletion of IT domain blocked EMD^WT‐FLAG^ and PDHA^WT‐HA^ interaction, whereas deletion of LEM domain did not have this effect (Figure 5H and S5D). In addition, we found that EMD could promote PDHA phosphorylation and interaction with PDK1, and deletion of IT domain abolished this effect (Figure 5I and S5E). Deletion of the 290–310 region (where S293 and S300 phosphorylation sites are located) inhibited the binding of PDHA to EMD, whereas deletion of the region 220–240 (where S232 phosphorylation site is located) did not affect the EMD‐PDHA interaction (Figure 5J and S5F). EMD‐PDHA interaction was inhibited by mutations of S293A and S300A, but not influenced by S232A mutation (Figure S5G). EMD inhibited the production of acetyl‐CoA catalysed by PDHA, resulted in an increase in the upstream pyruvate content, and these effects would be abolished by the deletion of IT domain (Figure 5K and S5H). However, PEP was not regulated by EMD (Figure 5K and S5G). In addition, PDHA S232A, S293A and S300A mutations did not influence EMD expression, indicating phosphorylation of PDHA had no effect on EMD (Figure 5L). These data suggested that IT domain of EMD and S293 and S300 phosphorylation of PDHA were essential for EMD and PDHA interaction.

### 3.8 EMD ISGylation was essential for aerobic oxidation inhibition

**FIGURE 6:**
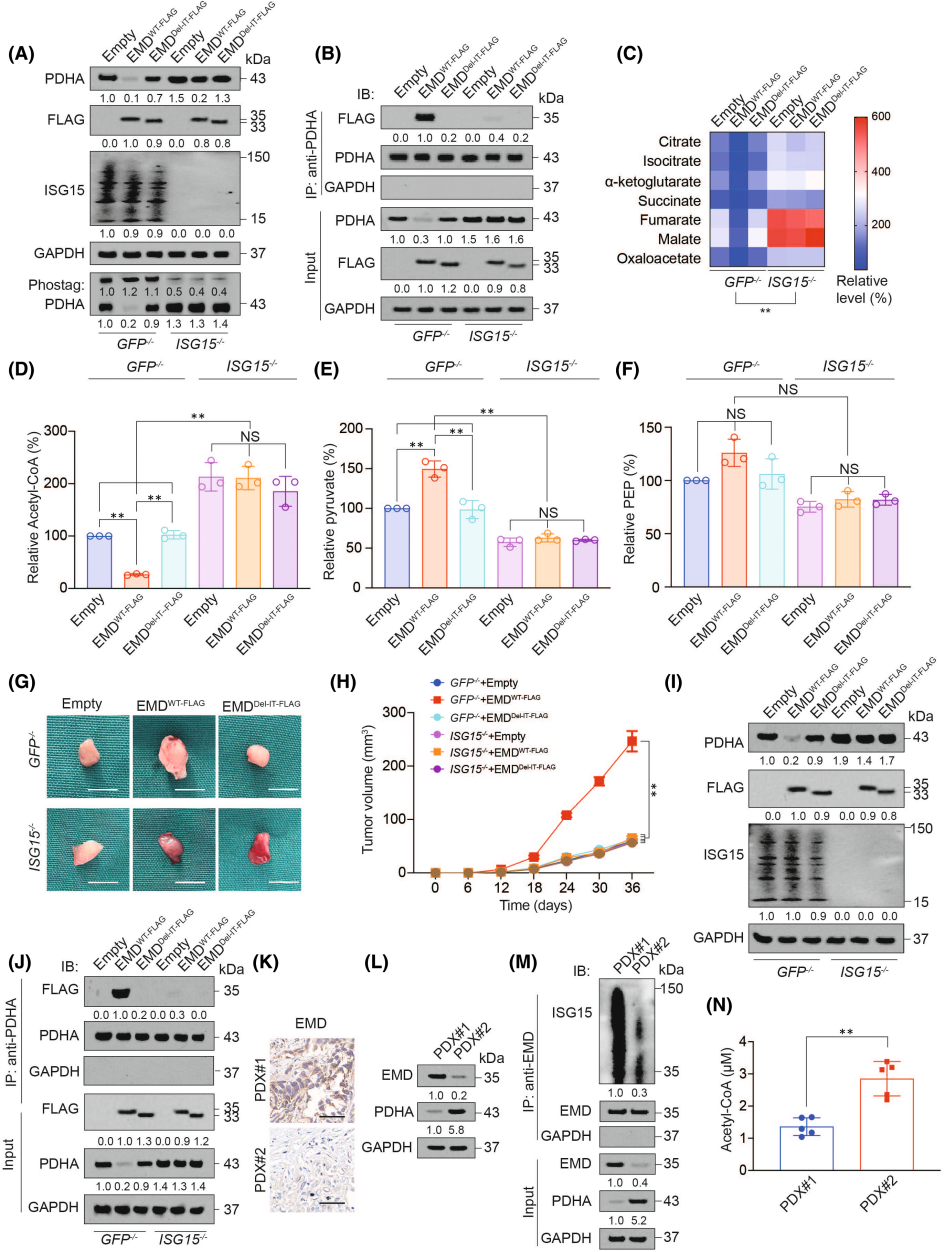
EMD ISGylation was essential for its inhibition on aerobic oxidation. (A) PDHA expression and phosphorylation, and global ISGylation were measured by IB in *GFP* ^*−/−*^ or *ISG15* ^*−/−*^ H2030 cells with or without EMD^WT‐FLAG^ or EMD^Del‐IT‐FLAG^ overexpression. (B) Co‐IP experiments were performed using anti‐PDHA antibodies in *GFP* ^*−/−*^ or *ISG15* ^*−/−*^ H2030 cells with or without EMD^WT‐FLAG^ or EMD^Del‐IT‐FLAG^ overexpression. Immunoprecipitated EMD‐FLAG was analysed by IB. The PDHA level in each co‐IP sample was adjusted to the same protein content. (C) Citrate, isocitrate, α‐ketoglutarate, succinate, fumarate, malate and oxaloacetate level were measured in *GFP* ^*−/−*^ or *ISG15* ^*−/−*^ H2030 cells with or without EMD^WT‐FLAG^ or EMD^Del‐IT‐FLAG^ overexpression. (D‐F) Relative acetyl‐CoA, pyruvate and PEP level were measured in *GFP* ^*−/−*^ or *ISG15* ^*−/−*^ H2030 cells with or without EMD^WT‐FLAG^ or EMD^Del‐IT‐FLAG^ overexpression. (G‐I) Representative images (G), tumour volume (H) and PDHA expression (I) for xenograft tumour formed by *GFP* ^*−/−*^ or *ISG15* ^*−/−*^ H2030 cells with or without EMD^WT‐FLAG^ or EMD^Del‐IT‐FLAG^ overexpression. Scale bar, 5 mm. (J) Co‐IP experiments were performed using anti‐PDHA to identify PDHA‐interacted EMD‐FLAG in xenograft tumour formed by *GFP* ^*−/−*^ or *ISG15* ^*−/−*^ H2030 cells with or without EMD^WT‐FLAG^ or EMD^Del‐IT‐FLAG^ overexpression. The PDHA level in each co‐IP sample was adjusted to the same protein content. (K) EMD expression measured by IHC in PDX model. Scale bar, 200 μm. (L) EMD and PDHA expressions measured by IB in PDX model. (M) EMD ISGylation measured by co‐IP in PDX model. The EMD level in each co‐IP sample was adjusted to the same protein content. (N) Acetyl‐CoA level in PDX model. The data are shown as the mean ± SD from three or five biological replicates (including IB). Data in C and H were analysed using a two‐way anova test. Data in D‐F were analysed using a one‐way anova test. Data in N were analysed by a Student's *t*‐test. **, *P* < 0.01, NS, non‐significant

Subsequently, we investigated that whether EMD ISGylation was related to its interaction with PDHA and regulation on aerobic oxidation. ISG15 knockout abolished the EMD inhibitory effect on PDHA expression and promotion effect on PDHA phosphorylation (Figure 6A and S6A) and disrupted the interaction between EMD and PDHA (Figure 6B and S6B). ISG15 knockout also abolished the suppression role of EMD on products of tricarboxylic acid cycle (Figure 6C and S6C) and acetyl‐CoA (Figure 6D and S6D), as well as the stimulation role of EMD on pyruvate (Figure 6E and S6E). However, ISG15 knockout did not significantly influence PEP level (Figure 6F and S6F). In xenograft formed by H2030 cells, we observed that deletion of IT domain or ISG15 knockout both abolished EMD stimulation on tumour growth (Figure 6G‐H), inhibition on PDHA expression (Figure 6I) and acetyl‐CoA level (Figure S6G), and blocked the interaction between EMD and PDHA (Figure 6J). These data elucidated the essential role of EMD ISGylation for its inhibition on PDHA and aerobic oxidation.

### 3.9 EMD was negatively associated with PDHA in PDX

We established two series of PDX and used immunohistochemistry (IHC) to identify that EMD expression was higher in PDX#1 compared with PDX#2 (Figure 6K). Interestingly, we found that PDHA expression was higher in PDX#2 compared with PDX#1 (Figure 6L). EMD level was also positively associated with its ISGylation level (Figure 6M) and negatively associated with acetyl‐CoA level (Figure 6N). The above data could further validate that EMD was negatively associated with PDHA and acetyl‐CoA, and positively associated with its ISGylation level.

### 3.10 Clinical correlation between EMD and PDHA

**FIGURE 7:**
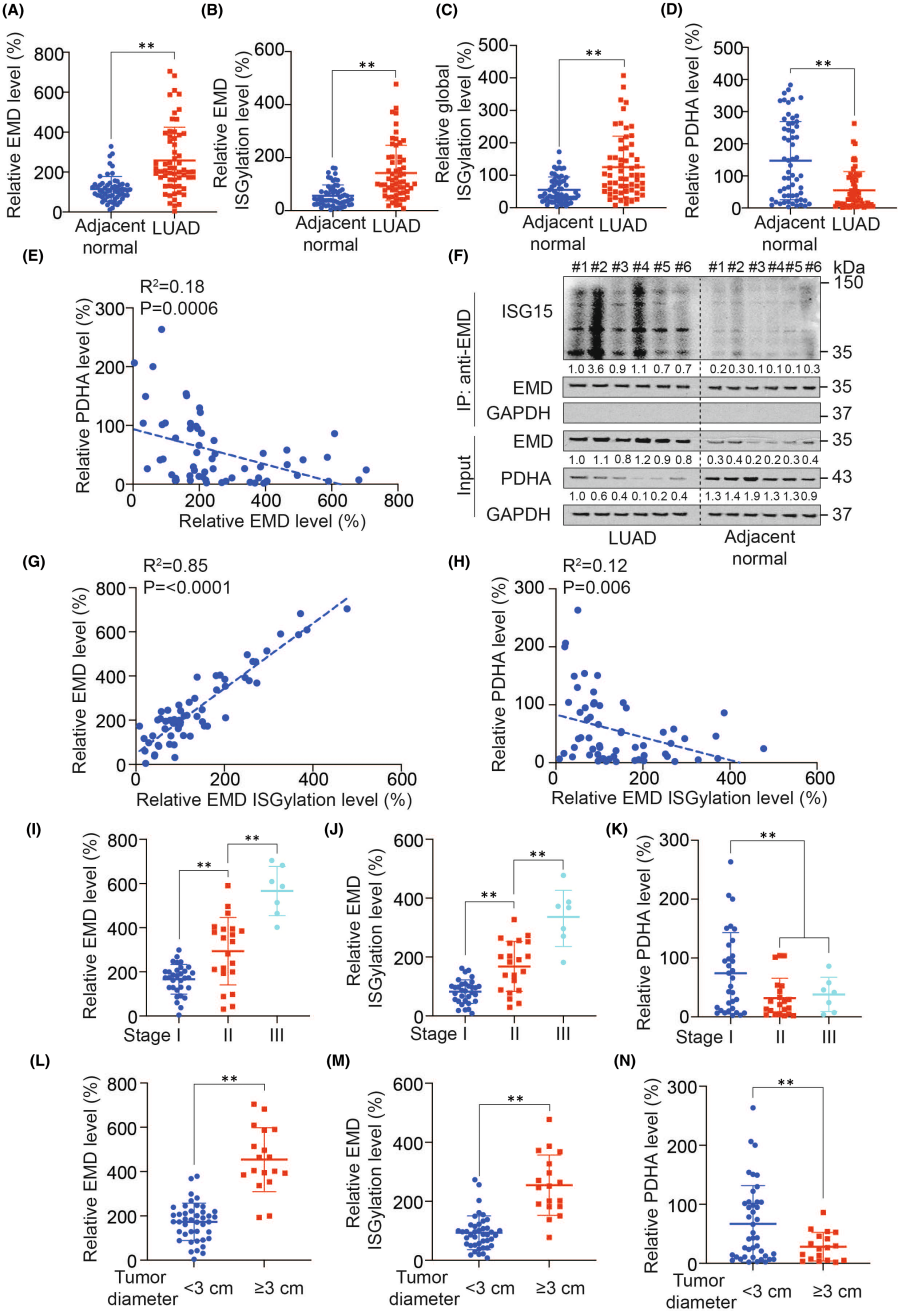
Clinical correlation between EMD and PDHA. (A‐D) EMD (A), EMD ISGylation (B), global ISGylation (C) and PDHA (D) level in LUAD and adjacent normal tissues (*n* = 60/group) from cohort #6. (E) Correlation between PDHA and EMD in LUAD tissues (*n* = 60/group) from cohort #6. (F) EMD expression and ISGylation in LUAD and adjacent normal tissues (*n* = 6/group) from cohort #6. The EMD level in each co‐IP sample was adjusted to the same protein content. (G) Correlation between EMD and its ISGylation in LUAD tissues (*n* = 60/group) from cohort #6. (H) Correlation between PDHA and EMD ISGylation in LUAD tissues (*n* = 60/group) from cohort #6. (I‐K) EMD (I), EMD ISGylation (J) and PDHA (K) level in stage I, II and III LUAD tissues from cohort #6. (L‐N) EMD (L), EMD ISGylation (M) and PDHA (N) level in tumour diameter <3 cm and ≥3 cm tissues from cohort #6. IB images were selected from three biological replicates. Data in A‐D and L‐N were analysed by a Student's *t*‐test. Data in E, G, H were analysed by a Spearman rank‐correlation analysis. Data in I‐K were analysed by a one‐way anova test. **, *P* < 0.01

After tested 60 paired of LUAD and adjacent normal tissues from cohort #6, we found that EMD, EMD ISGylation and global ISGylation raised, while PDHA declined in LUAD tissues compared with adjacent normal tissues (Figure 7A‐D), and EMD level was negatively correlated with PDHA level in LUAD tissues (Figure 7E). In addition, we found that EMD ISGylation was positively correlated with EMD (Figure 7F‐G) and global ISGylation level (Figure S7A), and negatively correlated with PDHA level (Figure 7H). The level of EMD and its ISGylation, as well as global ISGylation increased with the tumour stage progression (Figure 7I‐J, Figure S7B), whereas PDHA level decreased in stage II and III tissues compared with those of stage I (Figure 7K). Furthermore, EMD level, its ISGylation and global ISGylation was higher in LUAD tumours with ≥3 cm diameter (Figure 7L‐M, Figure S7C), whereas PDHA level was higher in LUAD tumours with <3 cm diameter (Figure 7N). Since EMD and PDHA were related with glucose metabolism in LUAD (Figure 5, 6).^21^ We evaluated that EMD expression, EMD ISGylation, global ISGylation and PDHA expression in high and low glucose LUAD tissues, and found that EMD expression, EMD ISGylation and global ISGylation increased, and PDHA expression decreased in high glucose tissues compared with low glucose tissues (Figure S7D‐H). These data suggested that EMD expression was positively correlated with its ISGylation, and negatively correlated with PDHA expression in clinical LUAD tissues.

## 4 DISCUSSION

EMD is an important integral protein of the nuclear inner membrane, but the mechanism that promotes its expression is not fully understood to a great extent. Existing studies confirmed that EMD transcription is controlled by transcription factor LIM domain 7,^36^ and its expression is inhibited by Semaphorin 3A.^37^ In this study, we explored that EMD expression was simultaneously controlled by ubiquitination and ISGylation. The ubiquitination occurred at K36 and ISGylation occurred at K37, and there was a dynamic balance between them, which controlled the expression of EMD; ubiquitination promoted the degradation of EMD, while ISGylation inhibited this process (Figure 2, 3). Given that EMD has more than 30 phosphorylation sites and more than five known O‐GlcNAcylated sites,^9^ whether phosphorylation and O‐GlcNAcylation would affect the ubiquitination and ISGylation of EMD, and even other post‐translational modifications are also involved in their dynamic balance, which can be further explored in subsequent studies. Based on the known high expression of EMD in breast cancer and ovarian epithelial cancer,^12^, ^13^ we further supplemented the evidence of high expression of EMD in LUAD and clarified the relevant mechanism to a certain extent. These results provide basis for individualized treatment of cancer.

The LEM‐domain is a ~ 40‐residue helix–loop–helix fold conserved both in eukaryotes and in prokaryotic DNA/RNA‐binding proteins.^38^ LEM domain in EMD can directly bind to lamins^39^ and barrier‐to‐autointegration factor (BAF),^40^ together forming a major component of nucleoskeletal structure known as the nuclear “lamina”.^41^ However, binding to PDHA was another domain of EMD, the IT domain (Figure 5). The IT domain also has the function to bind proteins and is known to bind β‐catenin.^8^ A deeper understanding of the properties of LEM and IT domains may lead to a more systematic understanding of the proteins they bind and their physiological effects. Followed by binding to PDHA, EMD promoted the phosphorylation and led to the degradation of PDHA (Figure 5), which is consistent with the previous study reported that PDHA phosphorylation leads to its inhibition.^22^

ISGylation modification is a kind of ubiquitin‐like modification, and both ISGylation and ubiquitination occurs at the K residue of the protein. Therefore, there are important connections between ISGylation and ubiquitination functions.^42^ ISGylation can inhibit ubiquitination by destroying the ubiquitin chain and inhibiting the connection between the target protein and the ubiquitination‐promoting protein, thereby inhibiting the degradation of the target protein.^16^, ^18^ Here, we found that ubiquitination and ISGylation of EMD occurred at adjacent sites (K36 and K37) and were completely existed (Figures 2 and 3). Therefore, this study proposed a previously unreported mechanism by which ISGylation affected ubiquitination‐related degradation. We speculated that there may be situations where ISGylation and ubiquitination compete for the same site, or ISGylation promotes ubiquitination at adjacent sites. These conjectures need to be further explored in follow‐up researches.

ISGylation is rarely induced under physiological conditions whereas abnormally elevated in various human diseases including several types of cancer.^43^ ISGylation can be induced in many patterns in cancer. Firstly, ISGylation can be induced by type I interferon (IFN) produced in the tumour microenvironment. Mechanistically, tumour‐derived HMGB1 induces the production of type I IFNs in dendritic cells via the TLR4‐MyD88 pathway, and tumour‐derived DNA activates the cGAS/STING pathway to drive the expression of type I IFNs through chaperoning HMGB1, autophagosome, exosome, LL37 or CLEC9A into dendritic cells.^44^ Studies also pointed out that IFN‐α promotes protein ISGylation in a SUMO3‐dependent manner through TRIM25, and enhanced IFNα‐induced holo‐ISGylation can also directly protect some proteins from proteasomal degradation.^45^ Secondly, miR‐2909, known to play a role in immunity and cancer, has been shown to upregulate ISGylation system through STAT1.^46^ Thirdly, in hepatocellular carcinoma tissues, enzymes involved in the ISGylation process (including *EFP*, *HERC5*, *UBA1* and *USP18*) are elevated compared with adjacent non‐tumour tissues.^47^ Fourthly, pancreatic cancer stem cell is reported to have the role to induce global ISGylation.^48^ Here, we demonstrated an increase in global ISGylation similar to other tumours in LUAD, and global ISGylation increase occurred in higher‐stage, larger‐diameter, higher‐glucose tumours.

Although EMD has been reported to be highly expressed in some types of tumours,^12^, ^13^ its tumour‐progression role has not been largely understood. In this study, we observed that the cancer‐promoting effect of EMD was achieved by binding and restricting the function of PDHA (Figures 5 and 6). Previous studies have clarified that downregulation of PDHA expression in tumours inhibits mitochondrial oxidative phosphorylation and global aerobic oxidation, promotes glycolysis, reduces reactive oxygen species production, enhances tumour cell malignancy and promotes tumour development.^49^, ^50^ In addition, low level of EMD is a key promoter of epithelial‐mesenchymal transition (EMT), for instance, by upregulating cytoplasmic p21.^51^ Therefore, low level of EMD has been reported to promote tumour metastasis.^52^ Why EMD plays opposite roles in tumour progression and metastasis? It would be important to determine whether EMD expression is different between metastatic subpopulations compared with the primary tumour. Here, we proposed a hypothesis that EMD induces hypoxia in tumours by inhibiting PDHA, and hypoxia can lead to activation of activating transcription factor 4 (ATF4), which maintains tumour cells in high levels of autophagy^53^; subsequently, EMD is degraded by autophagy, leading to the occurrence of pro‐invasive effects such as EMT. However, this hypothesis needs further experiments to validate. The possible dynamic changes of EMD poses a challenge to the treatment of tumours with high EMD expression. However, a deep understanding of EMD expression regulations and cancer‐promoting roles would provide a basis for the proposal of new tumour treatment strategies.

## AUTHOR CONTRIBUTIONS

**Congcong Zhang:** Data curation (equal); formal analysis (equal); investigation (equal); methodology (equal); validation (equal); visualization (equal); writing – original draft (equal). **Jiangtao Cui:** Data curation (equal); formal analysis (equal); investigation (equal); methodology (equal); validation (equal); visualization (equal). **Leiqun Cao:** Data curation (equal); formal analysis (equal); investigation (equal); methodology (equal); validation (equal); visualization (equal). **Xiaoting Tian:** Resources (lead). **Yayou Miao:** Resources (lead). **Shiyu Qiu:** Data curation (supporting); formal analysis (supporting); investigation (supporting); methodology (supporting); validation (supporting); visualization (supporting). **Yikun Wang:** Data curation (supporting); formal analysis (supporting); investigation (supporting); methodology (supporting); validation (supporting); visualization (supporting). **Wanxin Guo:** Data curation (supporting); formal analysis (supporting); investigation (supporting); methodology (supporting); validation (supporting); visualization (supporting). **Lifang Ma:** Conceptualization (equal); formal analysis (equal); funding acquisition (equal); project administration (equal); supervision (equal). **Jinjing Xia:** Conceptualization (equal); formal analysis (equal); funding acquisition (equal); project administration (equal); supervision (equal). **Xiao Zhang:** Conceptualization (equal); formal analysis (equal); funding acquisition (equal); project administration (equal); supervision (equal); writing – original draft (equal).

## CONFLICT OF INTEREST

The authors have no conflicts of interest to declare.

## Supporting information


**FIGURE S1** EMD expression in EMD.
(A‐C) EMD mRNA level was measured by qPCR in cohort #2 (A), #3 (B) and #4 (C).
(D‐E) Cell immunochemistry image (D) and its intensity (E). The intensity of A549 cell line was arbitrary set to 100%.
The data are shown as the mean ± SD from three biological replicates. Data in A‐C were analysed by a student’s *t* test. Data in E were analysed by a one‐way anova test. *, *P* < 0.05, **, *P* < 0.01.
**FIGURE S2**. EMD protein stability in LUAD cells.
(A) Protein intensity for Figure 2A. The protein intensities were normalized to those of GAPDH, and the intensity of EMD was arbitrary set to 100%.
(B‐C) Skeletal protein levels were measured by IB in H1299 cells treated with CHX (10 μg/ml) for indicated hours (B). The protein intensities were normalized to those of GAPDH, and the intensity of EMD was arbitrary set to 100% (C). (D) First BCA protein quantitation after elusion using the Acid Elution Buffer. (E) Second BCA protein quantitation after samples dilution according to the result of first BCA protein quantitation. (F) Colony formation were measured in H2030 and H1299 cells with or without EMD^WT‐FLAG^ or EMD^K36A‐FLAG^ overexpression. The images were shown in Figure 2D.
The data are shown as the mean ± SD from three biological replicates (including IB). Data in A and C were analysed using a two‐way anova test. Data in E and F were analysed using a one‐way anova test. *, *P* < 0.05, **, *P* < 0.01, NS, non‐significant.
**FIGURE S3**. EMD ISGylation in LUAD cells.
Co‐IP experiments using IgG for Figure 3B.
(B) The statistics for overlap of PSMB5 and EMD for Figure 3C (30 cells per group).
(C) Co‐IP experiments using IgG for Figure 3E.
(D) Co‐IP experiments using IgG and the input for Figure 3G.
(E) Co‐IP experiments using IgG for Figure 3J.
IB images were selected from three biological replicates. Data in B were analysed by a χ^2^ test.
**FIGURE S4**. EMD regulated glucose metabolism in LUAD cells.
(A) Intracellular glucose level in H2030 and H1299 cells with or without EMD overexpression or knockout.
(B) EMD expression measured by IB in H2030 and H1299 cells with or without EMD overexpression or knockout.
The data are shown as the mean ± SD from three biological replicates (including IB). Data in A were analysed by a student’s *t* test.
**FIGURE S5**. EMD interacted and suppressed PDHA in LUAD cells.
(A) PDHA and EMD expressions in H1299 cells measured by IB.
(B) mRNA level of aerobic oxidation and glycolysis related kinases was measured by qPCR in EMD overexpressed or knockout H1299 cells.
(C) Co‐IP experiments were performed using anti‐EMD or anti‐PDHA antibodies in H1299 cells. IgG‐immunoprecipitated or no‐antibody used samples were analysed in parallel. Co‐immunoprecipitated PDHA and EMD expression was measured.
(D) Reciprocal co‐IP experiments were performed using anti‐FLAG antibodies in PDHA‐HA overexpressed H1299 cells with or without EMD^WT‐FLAG^, EMD^Del‐LEM‐FLAG^ or EMD^Del‐IT‐FLAG^ overexpression.(E) PDHA expression and phosphorylation were measured by normal and Phostag‐IB respectively in H1299 cells with or without EMD^WT‐FLAG^, EMD^Del‐LEM‐FLAG^ or EMD^Del‐IT‐FLAG^ overexpression. PDHA and PDK1 interaction was measured by co‐IP using anti‐PDHA followed by IB experiments. The PDHA level in each co‐IP sample was adjusted to the same protein content. (F) co‐IP experiments were performed using anti‐HA antibodies in EMD overexpressed H1299 cells with or without PDHA^WT‐HA^, PDHA^Del‐220‐240‐HA^ or PDHA^Del‐290‐310‐HA^ overexpression.
(G) Reciprocal co‐IP experiments were performed using anti‐FLAG or anti‐HA antibodies in H1299 cells with or without indicated WT or mutant PDHA overexpressed. (H) Relative acetyl‐CoA, pyruvate and PEP level were measured in H1299 cells with or without EMD^WT‐FLAG^, EMD^Del‐LEM‐FLAG^ or EMD^Del‐IT‐FLAG^ overexpression. The data are shown as the mean ± SD from three biological replicates (including IB). Data in B and H were analysed using a one‐way anova test. **, *P* < 0.01, NS, non‐significant.
**FIGURE S6**. EMD ISGylation was essential for its inhibition on aerobic oxidation.(A) PDHA expression and global ISGylation were measured by IB in *GFP* ^*−/−*^ or *ISG15* ^*−/−*^ H1299 cells with or without EMD^WT‐FLAG^ or EMD^Del‐IT‐FLAG^ overexpression.(B) Co‐IP experiments were performed using anti‐PDHA antibodies in *GFP* ^*−/−*^ or *ISG15* ^*−/−*^ H1299 cells with or without EMD^WT‐FLAG^ or EMD^Del‐IT‐FLAG^ overexpression. Immunoprecipitated EMD‐FLAG was analysed by IB. The PDHA level in each co‐IP sample was adjusted to the same protein content.(C) Citrate, isocitrate, α‐ketoglutarate, succinate, fumarate, malate, and oxaloacetate level were measured in *WT* or *ISG15* ^*−/−*^ H1299 cells with or without EMD^WT‐FLAG^ or EMD^Del‐IT‐FLAG^ overexpression.(D‐F) Relative acetyl‐CoA, pyruvate and PEP level were measured in *GFP* ^*−/−*^ or *ISG15* ^*−/−*^ H1299 cells with or without EMD^WT‐FLAG^ or EMD^Del‐IT‐FLAG^ overexpression.(G) Relative acetyl‐CoA level was measured in xenograft tumour formed by *GFP* ^*−/−*^ or *ISG15* ^*−/−*^ H2030 cells with or without EMD^WT‐FLAG^ or EMD^Del‐IT‐FLAG^ overexpression.
The data are shown as the mean ± SD from three biological replicates (including IB). Data in C were analysed using a two‐way anova test. Data in D‐G were analysed using a one‐way anova test. **, *P* < 0.01, NS, non‐significant.
**FIGURE S7**. Clinical correlation between EMD and PDHA.
(A) Correlation between EMD ISGylation and global ISGylation in LUAD tissues (*n* = 60/group) from cohort #6.
(B‐C) Global ISGylation in stage I, II and III (B) and tumour diameter <3 cm and ≥3 cm (C) LUAD tissues from cohort #6.
(D‐G) EMD expression (D), EMD ISGylation (E), global ISGylation (F) and PDHA level (G) in low (<6.0 mM) or high (≥6.0 mM) glucose LUAD tissues from cohort #6.
(H) EMD expression, ISGylation and PDHA level analysed by IB in low (<6.0 mM) or high (≥6.0 mM) glucose LUAD tissues (*n* = 6/group) from cohort #6. The EMD level in each co‐IP sample was adjusted to the same protein content. Images were selected from three biological replicates.
Data in A were analysed by a Spearman rank‐correlation analysis. Data in B were analysed by a one‐way anova test. Data in C‐G were analysed by a student’s *t* test. **, *P* < 0.01.

**TABLE S1** Basic information for patient in cohort #1.
**TABLE S2**. Basic information for patient in cohort #2.
**TABLE S3**. Basic information for patient in cohort #3.
**TABLE S4**. Basic information for patient in cohort #4.
**TABLE S5**. Basic information for patient in cohort #5.
**TABLE S6**. Basic information for patient in cohort #6.
**TABLE S7**. Primers and sgRNAs used in the study.

## Data Availability

The data that support the findings of this study are available from the corresponding author upon reasonable request.
