# Supplementary material for: ISGylation of EMD promotes its interaction with PDHA to inhibit aerobic oxidation in lung adenocarcinoma
Source: J Cell Mol Med. 2022 Sep 7;26(19):5078–94. doi: 10.1111/jcmm.17536 (PMC9549505; doi:10.1111/jcmm.17536)
Supplement: Supplementary file 1 — FIGURE S1 EMD expression in EMD. (A‐C) EMD mRNA level was measured by qPCR in cohort #2 (A), #3 (B) and #4 (C). (D‐E) Cell immunochemistry image (D) and its intensity (E). The intensity of A549 cell line was arbitrary set to 100%. The data are shown as the mean ± SD from three biological replicates. Data in A‐C were analysed by a student’s t test. Data in E were analysed by a one‐way anova test. *, P < 0.05, **, P < 0.01. FIGURE S2. EMD protein stability in LUAD cells. (A) Protein intensity for Figure 2A. The protein intensities were normalized to those of GAPDH, and the intensity of EMD was arbitrary set to 100%. (B‐C) Skeletal protein levels were measured by IB in H1299 cells treated with CHX (10 μg/ml) for indicated hours (B). The protein intensities were normalized to those of GAPDH, and the intensity of EMD was arbitrary set to 100% (C). (D) First BCA protein quantitation after elusion using the Acid Elution Buffer. (E) Second BCA protein quantitation after samples dilution according to the result of first BCA protein quantitation. (F) Colony formation were measured in H2030 and H1299 cells with or without EMDWT‐FLAG or EMDK36A‐FLAG overexpression. The images were shown in Figure 2D. The data are shown as the mean ± SD from three biological replicates (including IB). Data in A and C were analysed using a two‐way anova test. Data in E and F were analysed using a one‐way anova test. *, P < 0.05, **, P < 0.01, NS, non‐significant. FIGURE S3. EMD ISGylation in LUAD cells. Co‐IP experiments using IgG for Figure 3B. (B) The statistics for overlap of PSMB5 and EMD for Figure 3C (30 cells per group). (C) Co‐IP experiments using IgG for Figure 3E. (D) Co‐IP experiments using IgG and the input for Figure 3G. (E) Co‐IP experiments using IgG for Figure 3J. IB images were selected from three biological replicates. Data in B were analysed by a χ2 test. FIGURE S4. EMD regulated glucose metabolism in LUAD cells. (A) Intracellular glucose level in H2030 and H1299 cells with or [file JCMM-26-5078-s002.docx]

**Figure S1**

**
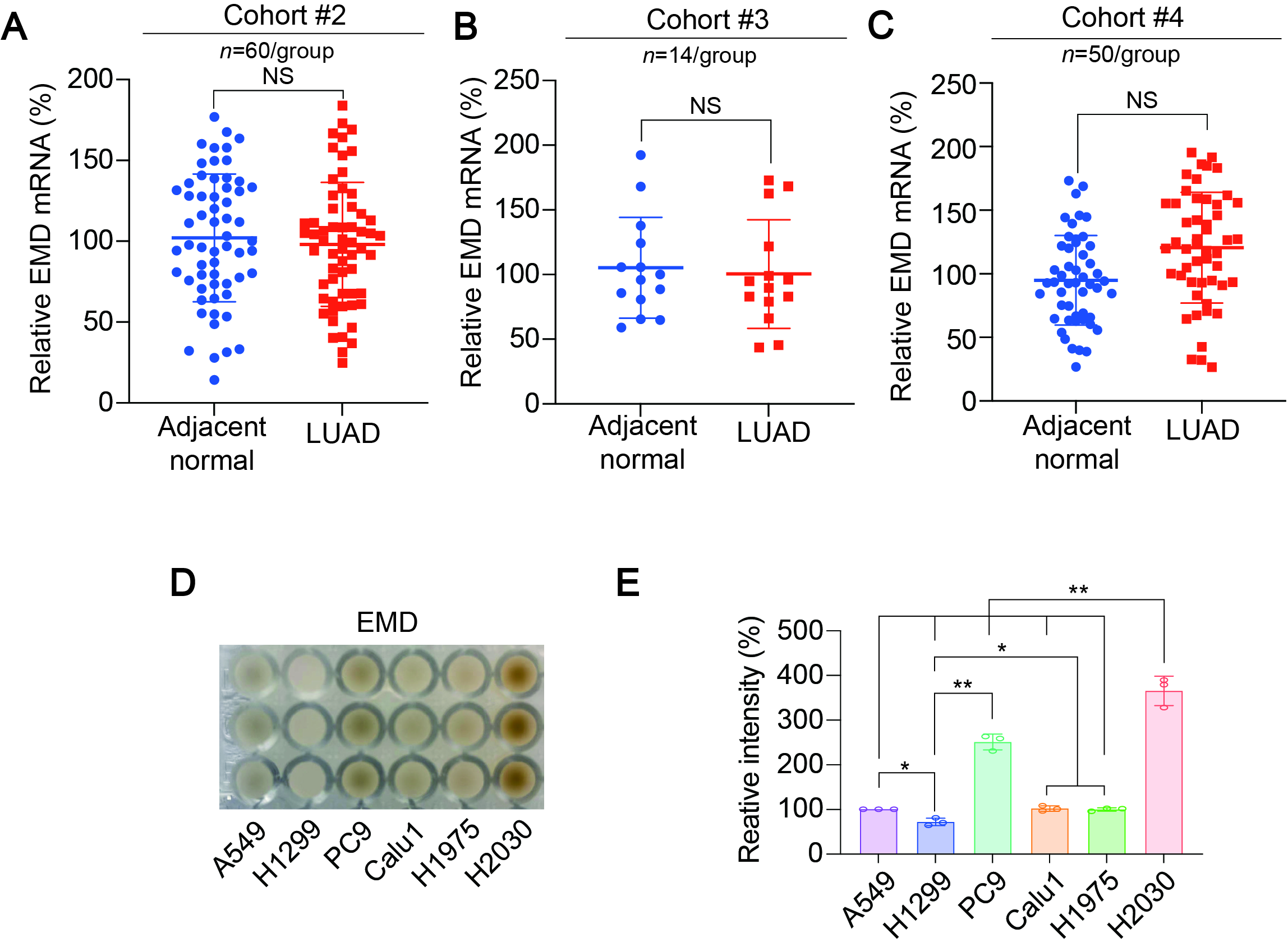
**



**Figure S1. EMD expression in EMD.**

(A-C) EMD mRNA level was measured by qPCR in cohort #2 (A), #3 (B) and #4 (C).

(D-E) Cell immunochemistry image (D) and its intensity (E). The intensity of A549 cell line was arbitrary set to 100%.

The data are shown as the mean ± SD from three biological replicates. Data in A-C were analyzed by a student’s t test. Data in E were analyzed by a one-way ANOVA test. *, P<0.05, **, P<0.01.

**Figure S2**


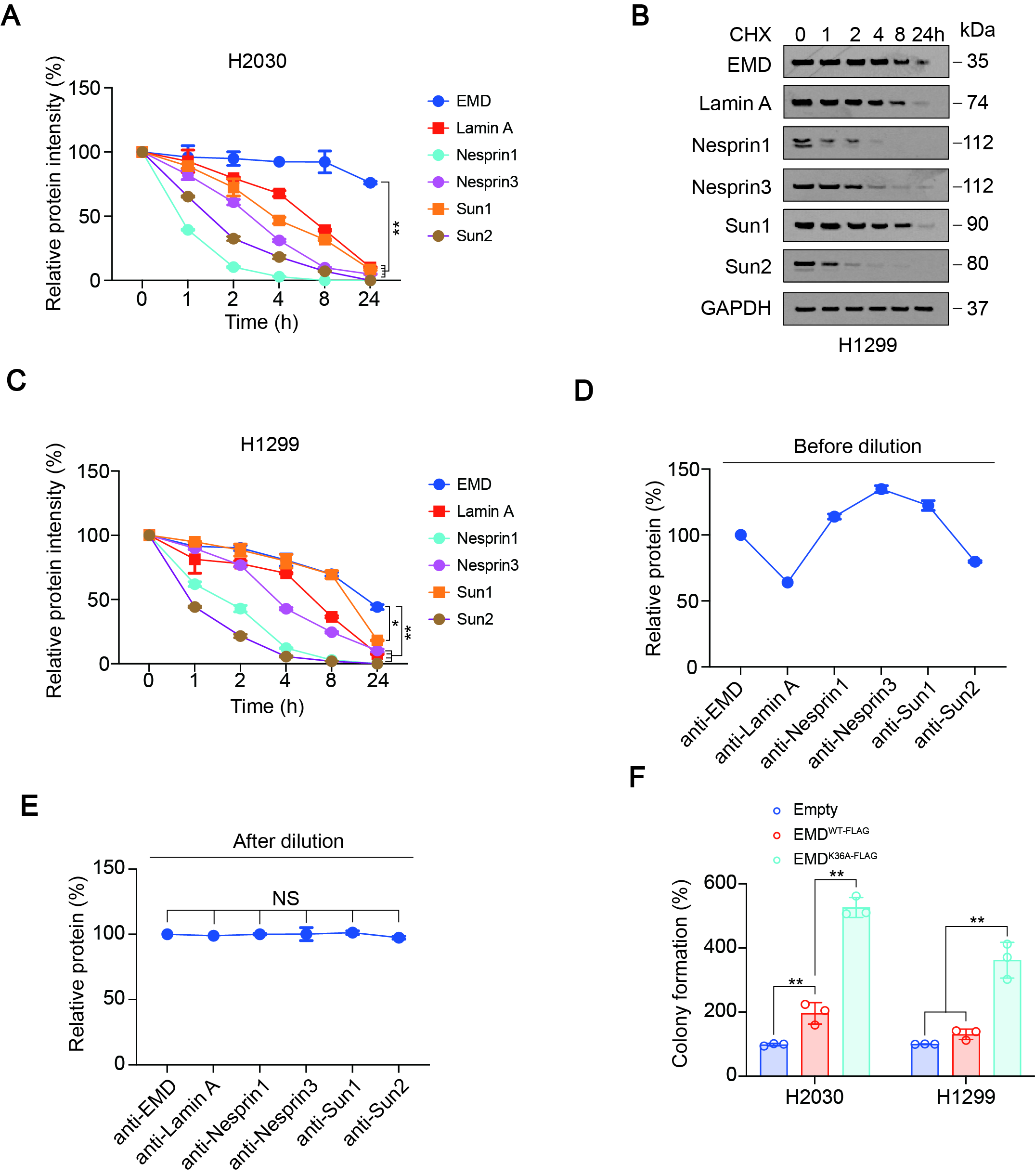


**Figure S2. EMD protein stability in LUAD cells.**

(A) Protein intensity for Figure 2A. The protein intensities were normalized to those of GAPDH, and the intensity of EMD was arbitrary set to 100%.

(B-C) Skeletal protein levels were measured by IB in H1299 cells treated with CHX (10 μg/ml) for indicated hours (B). The protein intensities were normalized to those of GAPDH, and the intensity of EMD was arbitrary set to 100% (C).

(D) First BCA protein quantitation after elusion using the Acid Elution Buffer.

(E) Second BCA protein quantitation after samples dilution according to the result of first BCA protein quantitation.

(F) Colony formation were measured in H2030 and H1299 cells with or without EMD^WT-FLAG^ or EMD^K36A-FLAG^ overexpression. The images were shown in Figure 2D.

The data are shown as the mean ± SD from three biological replicates (including IB). Data in A and C were analyzed using a two-way ANOVA test. Data in E and F were analyzed using a one-way ANOVA test. *, P<0.05, **, P<0.01, NS, non-significant.

**Figure S3**

**
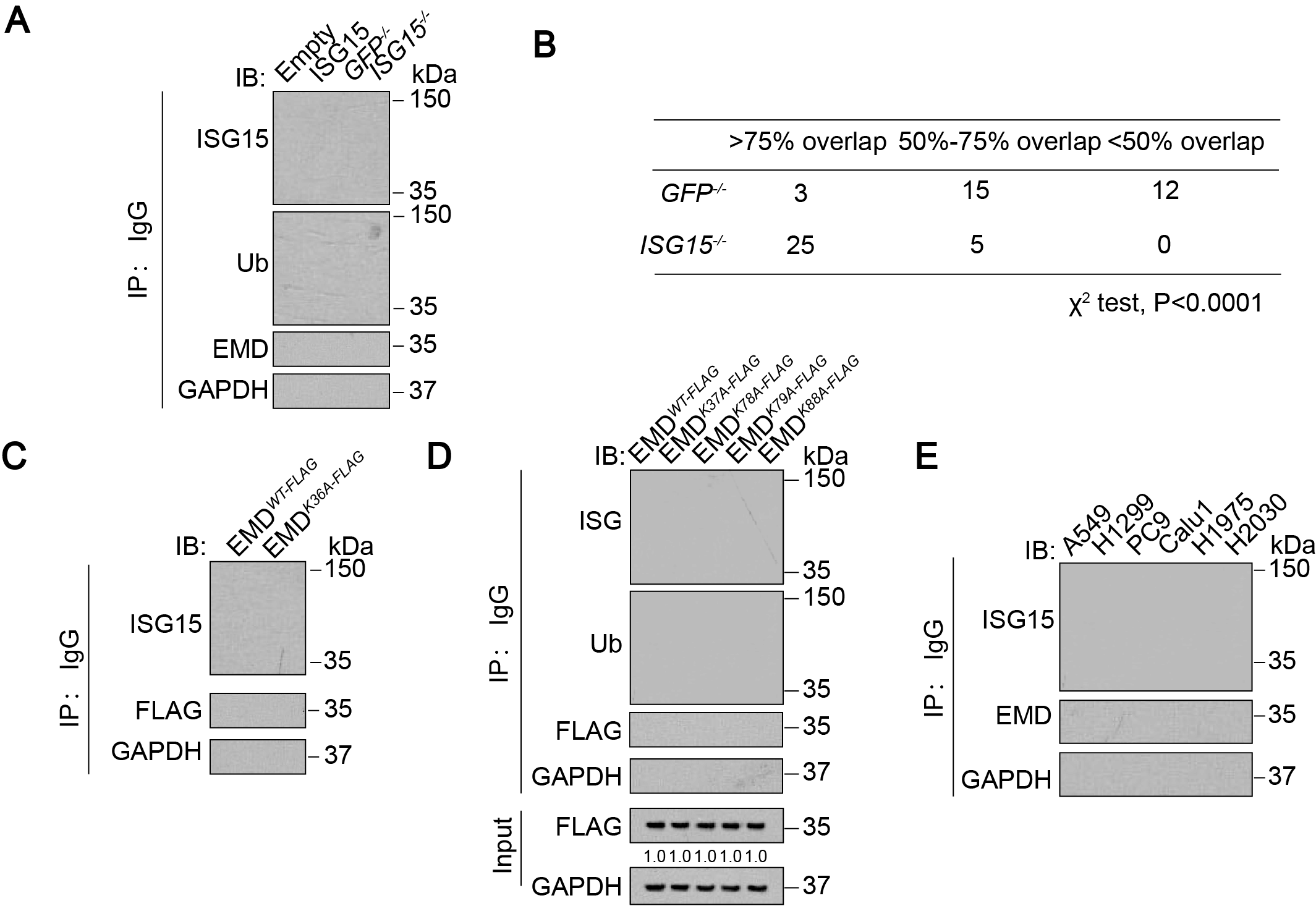
**

**Figure S3. EMD ISGylation in LUAD cells.**

1. Co-IP experiments using IgG for Figure 3B.

(B) The statistics for overlap of PSMB5 and EMD for Figure 3C (30 cells per group).

(C) Co-IP experiments using IgG for Figure 3E.

(D) Co-IP experiments using IgG and the input for Figure 3G.

(E) Co-IP experiments using IgG for Figure 3J.

IB images were selected from three biological replicates. Data in B were analyzed by a χ^2^ test.

**Figure S4**

**
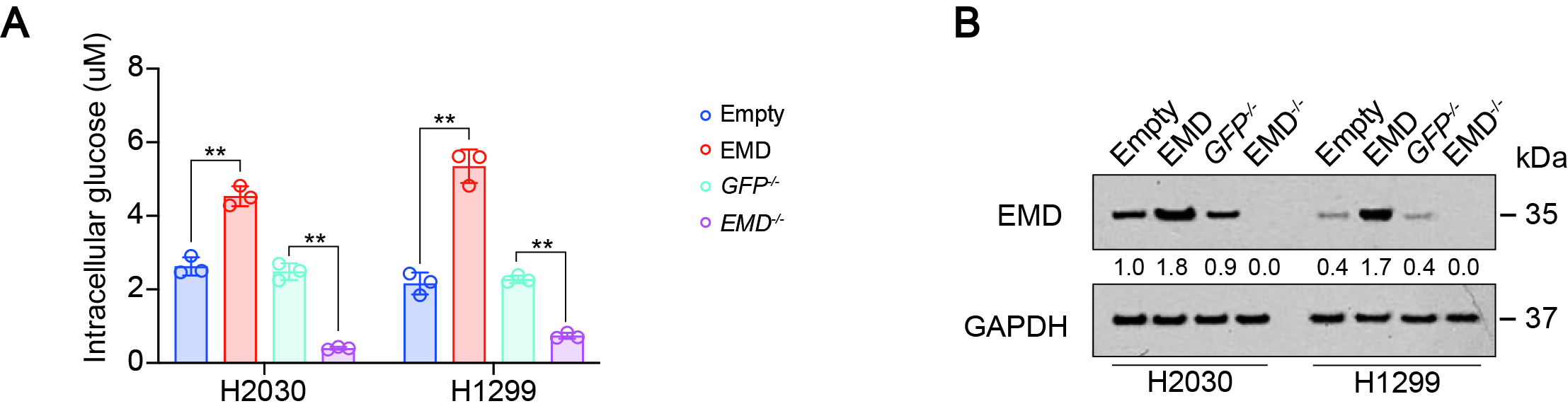
**

**Figure S4. EMD regulated glucose metabolism in LUAD cells.**

(A) Intracellular glucose level in H2030 and H1299 cells with or without EMD overexpression or knockout.

(B) EMD expression measured by IB in H2030 and H1299 cells with or without EMD overexpression or knockout.

The data are shown as the mean ± SD from three biological replicates (including IB). Data in A were analyzed by a student’s t test.

**Figure S5**

**
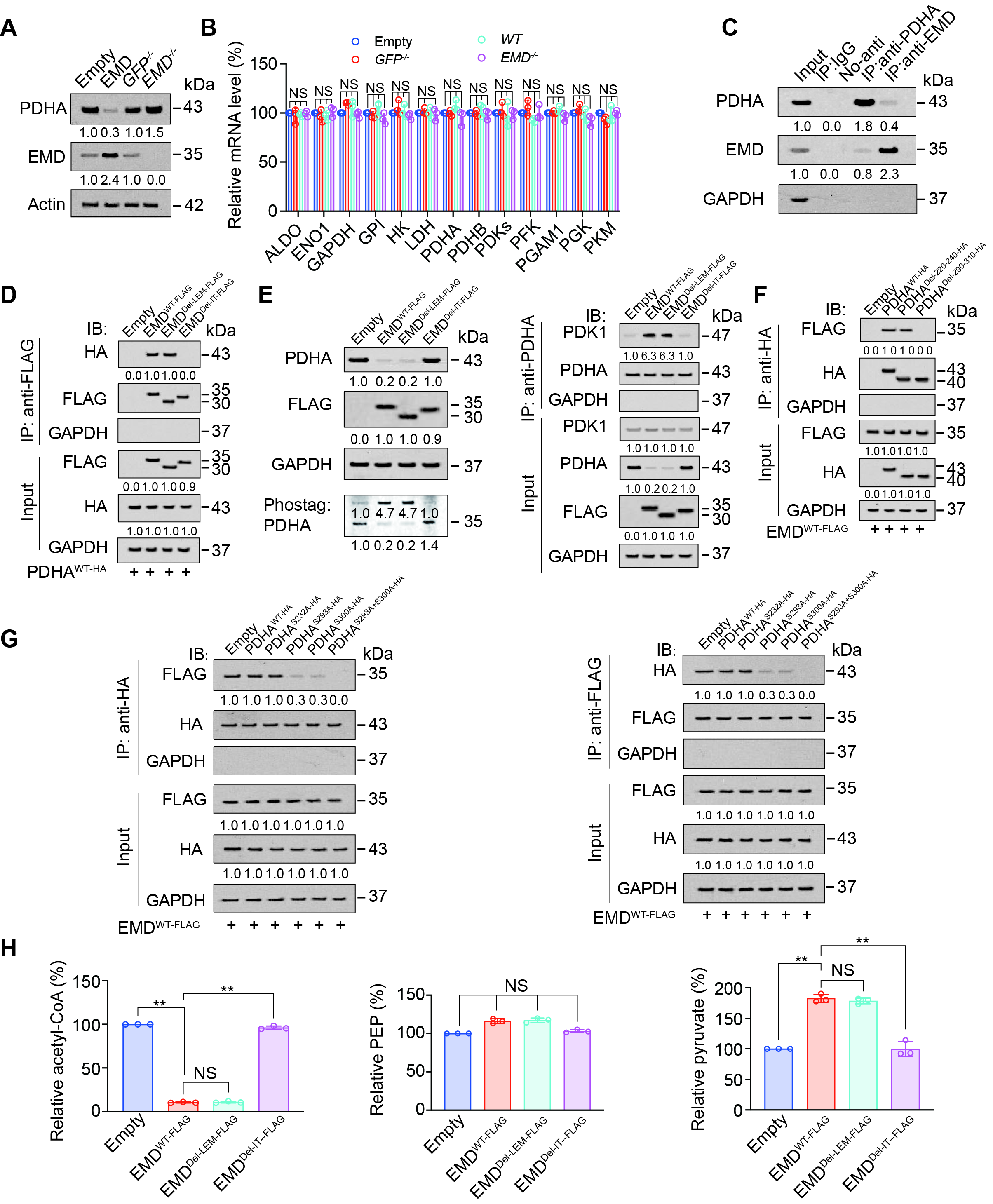
**

**Figure S5. EMD interacted and supppressed PDHA in LUAD cells.**

(A) PDHA and EMD expressions in H1299 cells measured by IB.

(B) mRNA level of aerobic oxidation and glycolysis related kinases was measured by qPCR in EMD overexpressed or knockout H1299 cells.

(C) Co-IP experiments were performed using anti-EMD or anti-PDHA antibodies in H1299 cells. IgG-immunoprecipitated or no-antibody used samples were analyzed in parallel. Co-immunoprecipitated PDHA and EMD expression was measured.

(D) Reciprocal co-IP experiments were performed using anti-FLAG antibodies in PDHA-HA overexpressed H1299 cells with or without EMD^WT-FLAG^, EMD^Del-LEM-FLAG^ or EMD^Del-IT-FLAG^ overexpression.

(E) PDHA expression and phosphorylation were measured by normal and Phostag-IB respectively in H1299 cells with or without EMD^WT-FLAG^, EMD^Del-LEM-FLAG^ or EMD^Del-IT-FLAG^ overexpression. PDHA and PDK1 interaction was measured by co-IP using anti-PDHA followed by IB experiments. The PDHA level in each co-IP sample was adjusted to the same protein content.

(F) co-IP experiments were performed using anti-HA antibodies in EMD overexpressed H1299 cells with or without PDHA^WT-HA^, PDHA^Del-220-240-HA^ or PDHA^Del-290-310-HA^ overexpression.

(G) Reciprocal co-IP experiments were performed using anti-FLAG or anti-HA antibodies in H1299 cells with or without indicated WT or mutant PDHA overexpressed.

(H) Relative acetyl-CoA, pyruvate and PEP level were measured in H1299 cells with or without EMD^WT-FLAG^, EMD^Del-LEM-FLAG^ or EMD^Del-IT-FLAG^ overexpression.

The data are shown as the mean ± SD from three biological replicates (including IB). Data in B and H were analyzed using a one-way ANOVA test. **, P<0.01, NS, non-significant.

**Figure S6**

**
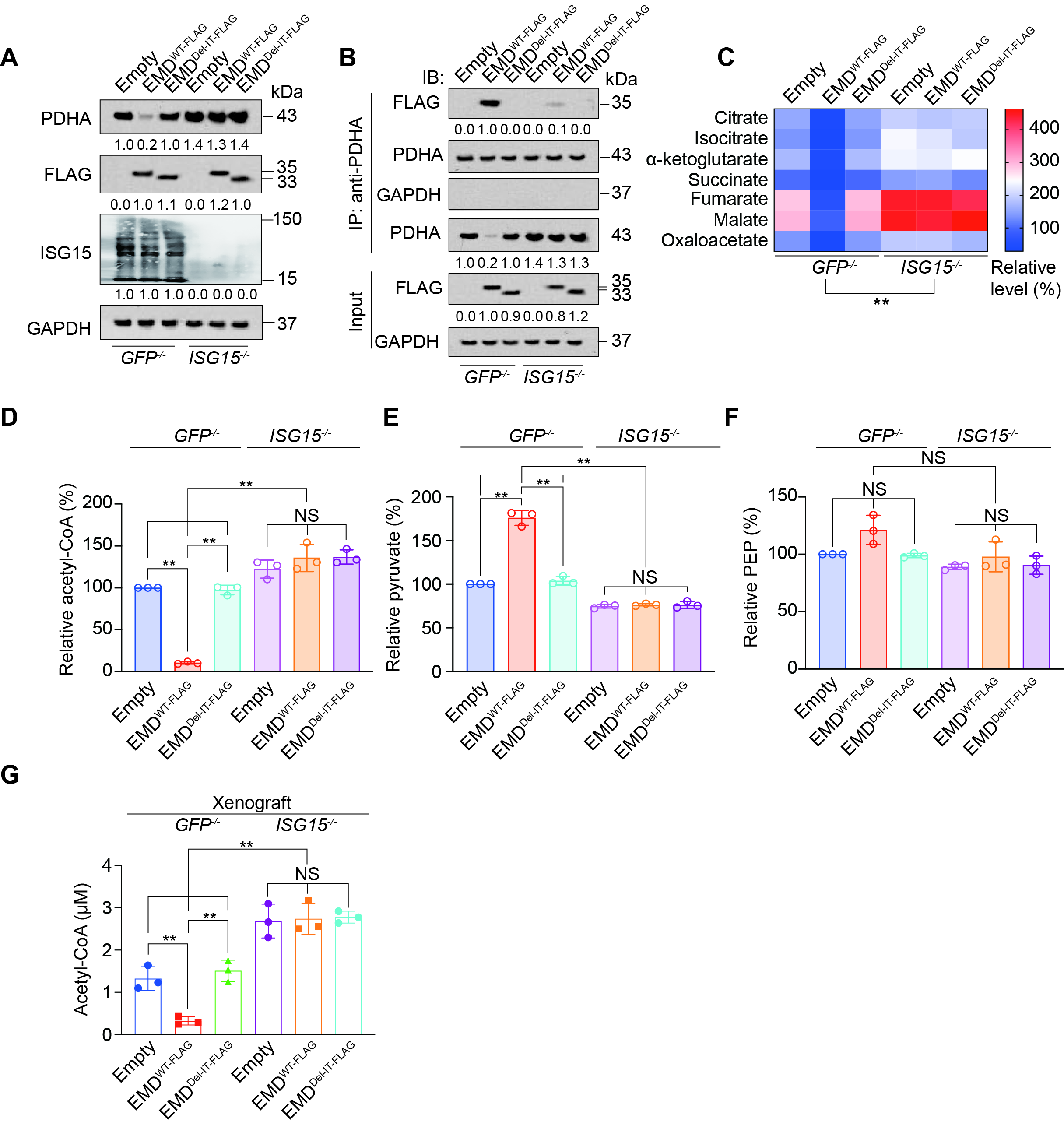
**

**Figure S6. EMD ISGylation was essential for its inhibition on aerobic oxidation.**

(A) PDHA expression and global ISGylation were measured by IB in *GFP^-/-^* or *ISG15^-/-^* H1299 cells with or without EMD^WT-FLAG^ or EMD^Del-IT-FLAG^ overexpression.

(B) Co-IP experiments were performed using anti-PDHA antibodies in *GFP^-/-^* or *ISG15^-/-^* H1299 cells with or without EMD^WT-FLAG^ or EMD^Del-IT-FLAG^ overexpression. Immunoprecipitated EMD-FLAG was analyzed by IB. The PDHA level in each co-IP sample was adjusted to the same protein content.

(C) Citrate, isocitrate, α-ketoglutarate, succinate, fumarate, malate, and oxaloacetate level were measured in *WT* or *ISG15^-/-^* H1299 cells with or without EMD^WT-FLAG^ or EMD^Del-IT-FLAG^ overexpression.

(D-F) Relative acetyl-CoA, pyruvate and PEP level were measured in *GFP^-/-^* or *ISG15^-/-^* H1299 cells with or without EMD^WT-FLAG^ or EMD^Del-IT-FLAG^ overexpression.

(G) Relative acetyl-CoA level was measured in xenograft tumor formed by *GFP^-/-^* or *ISG15^-/-^* H2030 cells with or without EMD^WT-FLAG^ or EMD^Del-IT-FLAG^ overexpression.

The data are shown as the mean ± SD from three biological replicates (including IB). Data in C were analyzed using a two-way ANOVA test. Data in D-G were analyzed using a one-way ANOVA test. **, P<0.01, NS, non-significant.

**Figure S7**

**
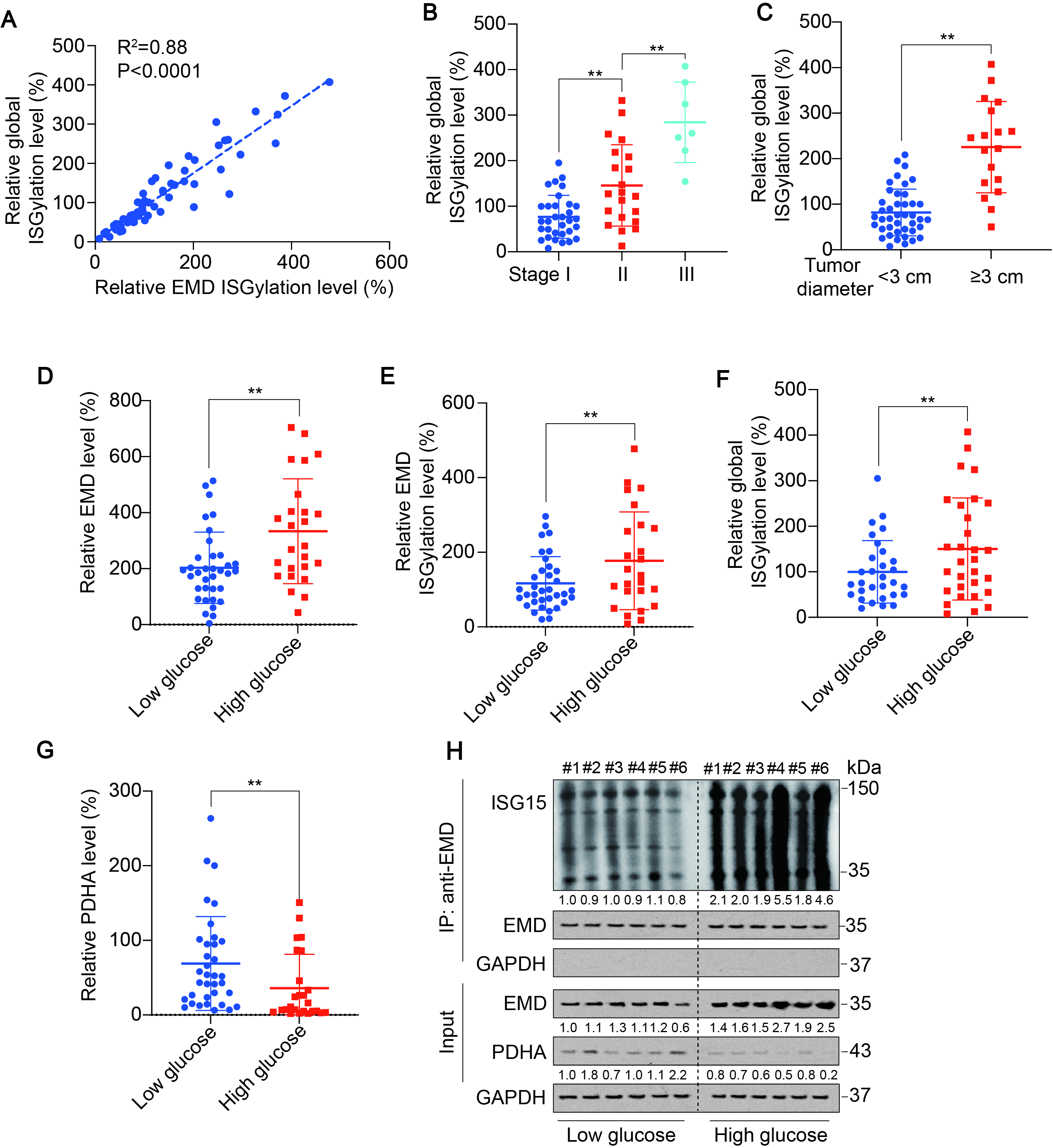
**

**Figure S7. Clinical correlation between EMD and PDHA.**

(A) Correlation between EMD ISGylation and global ISGylation in LUAD tissues (n=60/group) from cohort #6.

(B-C) Global ISGylation in stage I, II and III (B) and tumor diameter <3cm and ≥3cm (C) LUAD tissues from cohort #6.

(D-G) EMD expression (D), EMD ISGylation (E), global ISGylation (F) and PDHA level (G) in low (<6.0 mM) or high (≥6.0 mM) glucose LUAD tissues from cohort #6.

(H) EMD expression, ISGylation and PDHA level analyzed by IB in low (<6.0 mM) or high (≥6.0 mM) glucose LUAD tissues (n=6/group) from cohort #6. The EMD level in each co-IP sample was adjusted to the same protein content. Images were selected from three biological replicates.

Data in A were analyzed by a Spearman rank-correlation analysis. Data in B were analyzed by a one-way ANOVA test. Data in C-G were analyzed by a student’s t test. **, P<0.01.
