# Supplementary material for: ISGylation of EMD promotes its interaction with PDHA to inhibit aerobic oxidation in lung adenocarcinoma
Source: J Cell Mol Med. 2022 Sep 7;26(19):5078–94. doi: 10.1111/jcmm.17536 (PMC9549505; doi:10.1111/jcmm.17536)
Supplement: Supplementary file 2 — TABLE S1 Basic information for patient in cohort #1. TABLE S2. Basic information for patient in cohort #2. TABLE S3. Basic information for patient in cohort #3. TABLE S4. Basic information for patient in cohort #4. TABLE S5. Basic information for patient in cohort #5. TABLE S6. Basic information for patient in cohort #6. TABLE S7. Primers and sgRNAs used in the study. [file JCMM-26-5078-s001.docx]

**Table S1.** Basic information for patient in cohort #1.

| Normal (N=20） | | LUSC (N=20) | | LUAD (N=20) | |
| --- | --- | --- | --- | --- | --- |
| Age |  | Age |  | Age |  |
| ≥65 | 11 | ≥65 | 9 | ≥65 | 8 |
| <65 | 9 | <65 | 11 | <65 | 12 |
| Gender |  | Gender |  | Gender |  |
| Male | 11 | Male | 11 | Male | 11 |
| Female | 9 | Female | 9 | Female | 9 |
| Smoking |  | Smoking |  | Smoking |  |
| Yes | 6 | Yes | 5 | Yes | 8 |
| No | 14 | No | 15 | No | 12 |
| Stage |  | Stage |  | Stage |  |
| I | NA | I | 12 | I | 11 |
| II | NA | II | 5 | II | 6 |
| III | NA | III | 3 | III | 3 |
| Total | 20 | Total | 20 | Total | 20 |

**Table S2.** Basic information for patient in cohort #2.

| LUAD (n=60) |  |
| --- | --- |
| Age |  |
| ≥65 | 27 |
| <65 | 33 |
| Gender |  |
| Male | 32 |
| Female | 28 |
| Smoking |  |
| Yes | 19 |
| No | 41 |
| Stage |  |
| I | 27 |
| II | 19 |
| III | 14 |
| Total | 60 |

**Table S3.** Basic information for patient in cohort #3.

| LUAD (n=14） | |
| --- | --- |
| Age |  |
| ≥65 | 7 |
| <65 | 7 |
| Gender |  |
| Male | 8 |
| Female | 6 |
| Smoking |  |
| Yes | 5 |
| No | 9 |
| Stage |  |
| I | 7 |
| II | 5 |
| III | 2 |
| Total | 14 |

**Table S4.** Basic information for patient in cohort #4.

| LUAD (n=50) |  |
| --- | --- |
| Age |  |
| ≥65 | 23 |
| <65 | 27 |
| Gender |  |
| Male | 28 |
| Female | 22 |
| Smoking |  |
| Yes | 21 |
| No | 29 |
| Stage |  |
| I | 22 |
| II | 17 |
| III | 11 |
| Total | 50 |

**Table S5.** Basic information for patient in cohort #5.

| LUAD (N=193) |  |
| --- | --- |
| Age |  |
| ≥65 | 99 |
| <65 | 94 |
| Gender |  |
| Male | 99 |
| Female | 94 |
| Smoking |  |
| Yes | 88 |
| No | 105 |
| Stage |  |
| I | 65 |
| II | 67 |
| III | 61 |
| Total | 193 |

**Table S6.** Basic information for patient in cohort #6.

| LUAD (n=60) |  |
| --- | --- |
| Age |  |
| ≥65 | 27 |
| <65 | 33 |
| Gender |  |
| Male | 33 |
| Female | 27 |
| Smoking |  |
| Yes | 18 |
| No | 42 |
| Stage |  |
| I | 32 |
| II | 21 |
| III | 7 |
| Total | 60 |

**Table S7. Primers and sgRNAs used in the study.**

| Name | 5'-3' |
| --- | --- |
| EMD-qPCR-F | CCGCCTCCTCTTATAGCTTCT |
| EMD-qPCR-R | CTCTGGTAGAGTAAAGCGTCCT |
| ALDO-qPCR-F | ATGCCCTACCAATATCCAGCA |
| ALDO-qPCR-R | GCTCCCAGTGGACTCATCTG |
| ENO1-qPCR-F | AAAGCTGGTGCCGTTGAGAA |
| ENO1-qPCR-R | GGTTGTGGTAAACCTCTGCTC |
| GAPDH-qPCR-F | GGAGCGAGATCCCTCCAAAAT |
| GAPDH-qPCR-R | GGCTGTTGTCATACTTCTCATGG |
| GPI-qPCR-F | CAAGGACCGCTTCAACCACTT |
| GPI-qPCR-R | CCAGGATGGGTGTGTTTGACC |
| HK-qPCR-F | GCTCTCCGATGAAACTCTCATAG |
| HK-qPCR-R | GGACCTTACGAATGTTGGCAA |
| LDH-qPCR-F | ATGGCAACTCTAAAGGATCAGC |
| LDH-qPCR-R | CCAACCCCAACAACTGTAATCT |
| PDHA-qPCR-F | TGGTAGCATCCCGTAATTTTGC |
| PDHA-qPCR-R | ATTCGGCGTACAGTCTGCATC |
| PDHB-qPCR-F | AAGAGGCGCTTTCACTGGAC |
| PDHB-qPCR-R | ACTAACCTTGTATGCCCCATCA |
| PDK1-qPCR-F | CTGTGATACGGATCAGAAACCG |
| PDK1-qPCR-R | TCCACCAAACAATAAAGAGTGCT |
| PDK2-qPCR-F | ATGAAAGAGATCAACCTGCTTCC |
| PDK2-qPCR-R | GGCTCTGGACATACCAGCTC |
| PDK3-qPCR-F | CGCTCTCCATCAAACAATTCCT |
| PDK3-qPCR-R | CCACTGAAGGGCGGTTAAGTA |
| PFK-qPCR-F | AGAAGGGGCTCATCCATACCC |
| PFK-qPCR-R | CTCTCGTCGATACTGGCCTAA |
| PGAM1-qPCR-F | GTGCAGAAGAGAGCGATCCG |
| PGAM1-qPCR-R | CGGTTAGACCCCCATAGTGC |
| PGK-qPCR-F | TGGACGTTAAAGGGAAGCGG |
| PGK-qPCR-R | GCTCATAAGGACTACCGACTTGG |
| PKM-qPCR-F | ATGTCGAAGCCCCATAGTGAA |
| PKM-qPCR-R | TGGGTGGTGAATCAATGTCCA |
| Actin-qPCR-F | CATGTACGTTGCTATCCAGGC |
| Actin-qPCR-R | CTCCTTAATGTCACGCACGAT |
| EMD-K36A-FLAG-F | AAGATCTTCGAGTACGAGACCCAGA |
| EMD-K36A-FLAG-R | CGTACTCGAAGATCTTAGCCTCGTAAAGCCTACGAGTT |
| EMD-K37A-FLAG-F | ATCTTCGAGTACGAGACCCAGAGGC |
| EMD-K37A-FLAG-R | TCGTACTCGAAGATAGCCTTCTCGTAAAGCCTACGAGTTG |
| EMD-K78A-FLAG-F | AAAGAGGACGCTTTACTCTACCAGAG |
| EMD-K78A-FLAG-R | AAAGCGTCCTCTTTAGCGGGAAGATCATACATATCTGCAT |
| EMD-K79A-FLAG-F | GAGGACGCTTTACTCTACCAGAGCA |
| EMD-K79A-FLAG-R | GTAGAGTAAAGCGTCCTCAGCCTTGGGAAGATCATACATA |
| EMD-K88A-FLAG-F | GGCTACAATGACGACTACTATGAAG |
| EMD-K88A-FLAG-R | GTCATTGTAGCCAGCGCTCTGGTAGAGTAAAGCGTCCTCT |
| EMD-Del-LEM-FLAG-F | TTAAGGTACCATGCGGCGGCTCTCGCCCCCCAGCTCGT |
| EMD-Del-LEM-FLAG-R | ATCGGCGGCCGCCTACTTGTCATCGTCGTCCTTGTAATCGAAGGGGTTGCCTTCTTCAGCCTGC |
| EMD-Del-IT-FLAG-F | TCCACCTCTTTTATGTCCTCCTCAT |
| EMD-Del-IT-FLAG-R | GGACATAAAAGAGGTGGAGTAGTGCGTGATGCTCTGGTAG |
| EMD-WT-FLAG-F | ATCGGGTACCATGGACAACTACGCAGATCTTTCGG |
| EMD-WT-FLAG-R | ATCGGCGGCCGCCTACTTGTCATCGTCGTCCTTGTAATCGAAGGGGTTGCCTTCTTCAGCCTGC |
| PDHA-Del-220-240-HA-F | GATTACTACAAGAGAGGCGATTTC |
| PDHA-Del-220-240-HA-R | TCTCTTGTAGTAATCAATACAAGGTAATTTCCACAAAGCT |
| PDHA-Del-290-310-HA-F | AGAAGTAAGAGTGACCCTATTATGCTTC |
| PDHA-Del-290-310-HA-R | GTCACTCTTACTTCTGTAACGGTAAGTCTGCAGCTCCATC |
| PDHA-S232A-HA-F | GTTGAGAGAGCGGCAGCCAGCACTG |
| PDHA-S232A-HA-R | CTCTCTCAACGGCCGTTCCCATTCCATAGCGATTATTCTC |
| PDHA-S293A-HA-F | ATGAGTGACCCTGGAGTCAGTTACC |
| PDHA-S293A-HA-R | AGGGTCACTCATGGCGTGTCCGTGGTAACGGTAAGTCTGCA |
| PDHA-S300A-HA-F | TACCGTACACGAGAAGAAATTCAGG |
| PDHA-S300A-HA-R | TTCTTCTCGTGTACGGTAGGCGACTCCAGGGTCACTCATA |
| WT-PDHA-HA-F | GTACGGTACCATGAGGAAGATGCTCGCCGCCGTCT |
| WT-PDHA-HA-R | GTACGCGGCCGCTTAAGCGTAGTCTGGGACGTCGTATGGGTAACTGACTGACTTAAACTTGATCCAC |
| EMD-sgRNA | GATGTTGTACCGGCGCAGCA |
